# Changes in alcohol use and mood during the COVID-19 pandemic among individuals with traumatic brain injury: A difference-in-difference study

**DOI:** 10.1371/journal.pone.0266422

**Published:** 2022-04-07

**Authors:** Raj G. Kumar, Dmitry Esterov, Rachel Sayko Adams, John D. Corrigan, Shannon B. Juengst, Nancy D. Chiaravalloti, Belinda Yew, Laura E. Dreer, Kristen Dams-O’Connor

**Affiliations:** 1 Department of Rehabilitation & Human Performance, Icahn School of Medicine at Mount Sinai, New York, NY, United States of America; 2 Department of Physical Medicine and Rehabilitation, Mayo Clinic, Rochester, MN, United States of America; 3 Institute for Behavioral Health, The Heller School for Social Policy and Management, Brandeis University, Waltham, Massachusetts, United States of America; 4 VHA Rocky Mountain Mental Illness Research Education and Clinical Center, Aurora, Colorado, United States of America; 5 Department of Physical Medicine and Rehabilitation, The Ohio State University, Columbus, OH, United States of America; 6 TIRR Memorial Hermann, Houston TX, United States of America; 7 Department of Physical Medicine & Rehabilitation, UT Southwestern, Dallas, TX, United States of America; 8 Kessler Foundation, East Hanover, NJ, United States of America; 9 Department of Physical Medicine and Rehabilitation, Rutgers University/The State University of New Jersey, New Brunswick, NJ, United States of America; 10 Department of Ophthalmology & Visual Sciences, University of Alabama at Birmingham, Birmingham, AL, United States of America; 11 Department of Physical Medicine & Rehabilitation, University of Alabama at Birmingham, Birmingham, AL, United States of America; 12 Department of Neurology, Icahn School of Medicine at Mount Sinai, New York, NY, United States of America; Yale School of Medicine, UNITED STATES

## Abstract

**Objective:**

To evaluate the impact of COVID-19 pandemic exposure on changes in alcohol use and mood from years 1 to 2 after traumatic brain injury (TBI).

**Methods:**

We used a difference-in-difference (DiD) study design to analyze data from 1,059 individuals with moderate-to-severe TBI enrolled in the TBI Model Systems (TBIMS) National Database. We defined COVID-19 pandemic exposure as participants who received their year 1 post-injury interviews prior to January 1, 2020, and their year 2 interview between April 1, 2020 and January 15, 2021. Pandemic-unexposed participants had both year 1 and 2 follow-up interviews before January 1, 2020. We measured current alcohol use as any past month alcohol use, average number of drinks per drinking occasion, and past month binge drinking. We measured depression symptoms using Patient Health Questionnaire-9, and anxiety symptoms using the Generalized Anxiety Disorder-7.

**Results:**

We found persons with TBI exposed to the pandemic had greater increases in the average number of drinks per occasion from year 1 to 2 post-injury compared to pandemic-unexposed individuals (β = 0.36, 95% CI: 0.16, 0.57, p = 0.001), with males, adults <65 years old, and Black and Hispanic subgroups showing the greatest increases in consumption. Though average consumption was elevated, changes in rates of any alcohol use or binge drinking by pandemic exposure were not observed. Overall, there were no significant changes in depressive and anxiety symptoms over time between pandemic exposed and unexposed groups; however, pandemic-exposed Hispanics with TBI reported significant increases in anxiety symptoms from year-1 to year-2 post-injury compared to pandemic-unexposed Hispanics (β = 2.35, 95% CI: 0.25, 4.47, p = 0.028).

**Conclusion:**

Among persons living with TBI, those exposed to the pandemic had significant increases in average alcohol consumption. Pandemic-exposed Hispanics with TBI had large elevations in anxiety symptoms, perhaps reflecting health inequities exacerbated by the pandemic, and suggesting a need for targeted monitoring of psychosocial distress.

## Introduction

The coronavirus disease-2019 (COVID-19) pandemic has resulted in widespread societal consequences, including death, strained healthcare systems, and tremendous economic disruption. In addition to the direct sequelae from infection, more people worldwide are reporting increased psychological distress [1, 2] and alcohol use [3, 4] during the pandemic. Studies of U.S. populations have found elevated distress [5], with up to a third of individuals meeting criteria for depressive or anxiety disorders during the pandemic [6, 7]. Moreover, greater psychological distress has been associated with more alcohol use [8], with correlations found between increased binge drinking during the pandemic and concomitant depressive symptoms [9].

Individuals with disabilities have historically faced barriers to accessing healthcare, resulting in health inequity, particularly among racial/ethnic minority groups [10]. The COVID-19 pandemic has magnified these longstanding inequities [11]; persons with disabilities are at higher risk of serious illness secondary to underlying neurologic conditions [12, 13] and have more difficulty engaging in COVID-19 preventative measures [14]. Persons with traumatic brain injury (TBI) represent an estimated 11.4 million persons with disability [15] who may be particularly affected by the pandemic, though currently only one cross-sectional study exists on how the COVID-19 pandemic has affected TBI survivors [16].

People who have incurred a TBI experience higher rates of depression [17–19] and anxiety [20–22] relative to individuals without a TBI. One large TBI study found cumulative prevalence of any psychiatric diagnosis (including depression, anxiety, and substance use disorders) was between 30–50% [23]. Presence of depression, anxiety, and/or at-risk substance use (including alcohol) has been linked to poorer physical, cognitive, and functional outcomes post-TBI [24–26].

It is unknown if the COVID-19 pandemic has exacerbated at-risk alcohol use or mood disorders among individuals with TBI. Given the high base rates of alcohol use and mood disorders among persons with TBI, distinction between “typical” TBI trajectory and pandemic-induced changes is necessary for any causal interpretation of the impact of the pandemic. Using a difference-in-difference (DiD) analytic approach, the present study aimed to elucidate the impact of the COVID-19 pandemic on alcohol use, depressive and anxiety symptoms among individuals living with TBI. In addition, given emerging evidence of health disparities [27, 28] and racial/ethnic differences in alcohol use and psychological distress during the pandemic [4, 5, 29], we evaluated whether the pandemic differentially affected demographic subgroups with TBI.

## Methods

### Participants

We drew our sample from the Traumatic Brain Injury Model Systems (TBIMS) National Database. This longitudinal database follows persons with a moderate-to-severe TBI at 1, 2, 5, and every subsequent 5 years after injury until death, 16+ years old, have sustained a moderate or severe TBI, and received inpatient rehabilitation at a TBIMS center. TBIMS participants provide informed consent directly or by proxy, and the study is overseen by each TBIMS center’s institutional review board.

**Fig 1:**
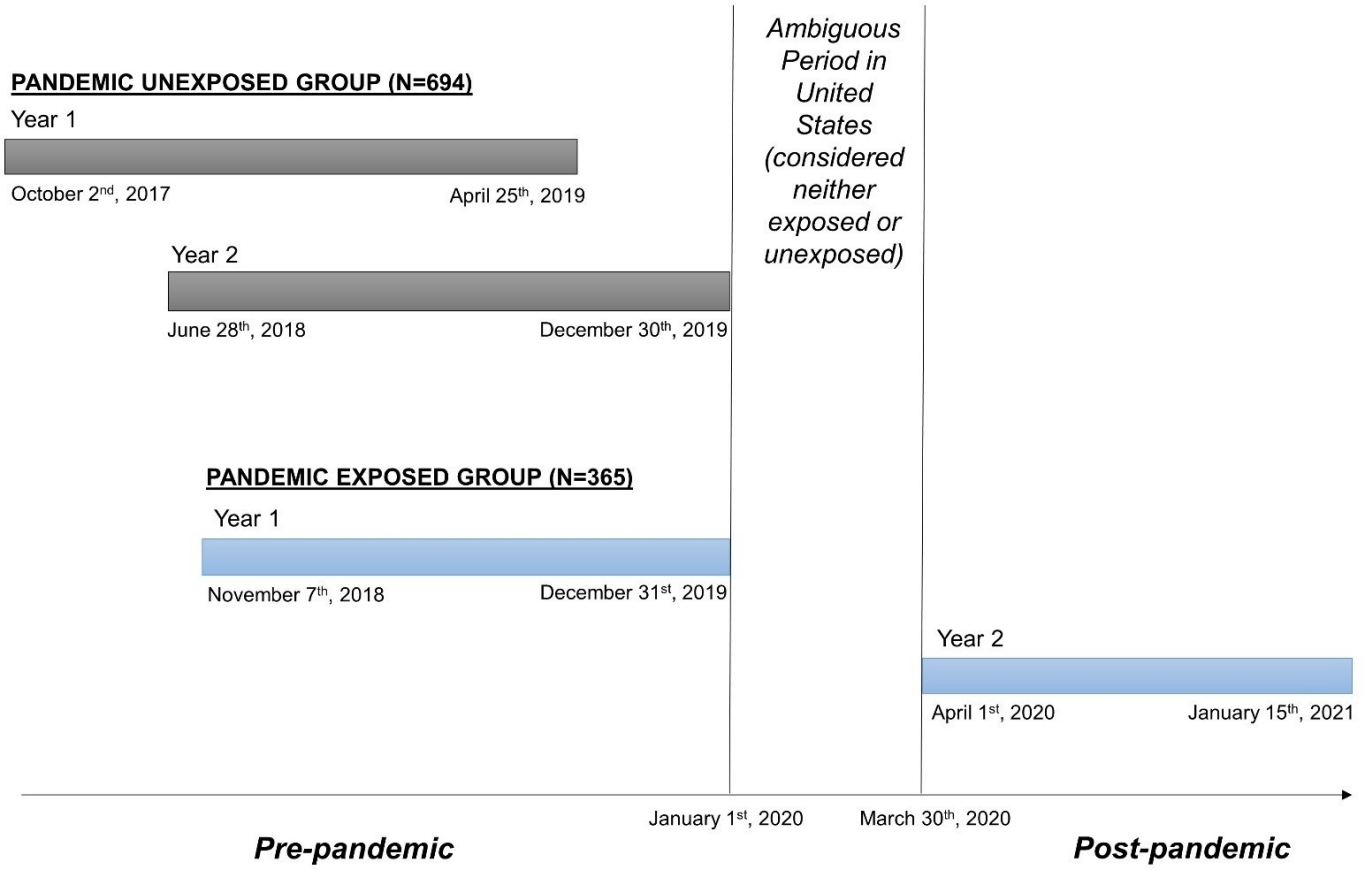
Timeline of COVID-19 pandemic exposure groups. We created two, mutually-exclusive pandemic exposure groups from Y1 and Y2 post-TBI data from the TBIMS National Database. The pandemic unexposed group had both their Y1 and Y2 interviews prior to January 1, 2020. While, the pandemic exposed group had their Y1 interview prior to January 1, 2020, and their Y2 interview between April 1^st^, 2020 and January 15^th^, 2021.

**Fig 2:**
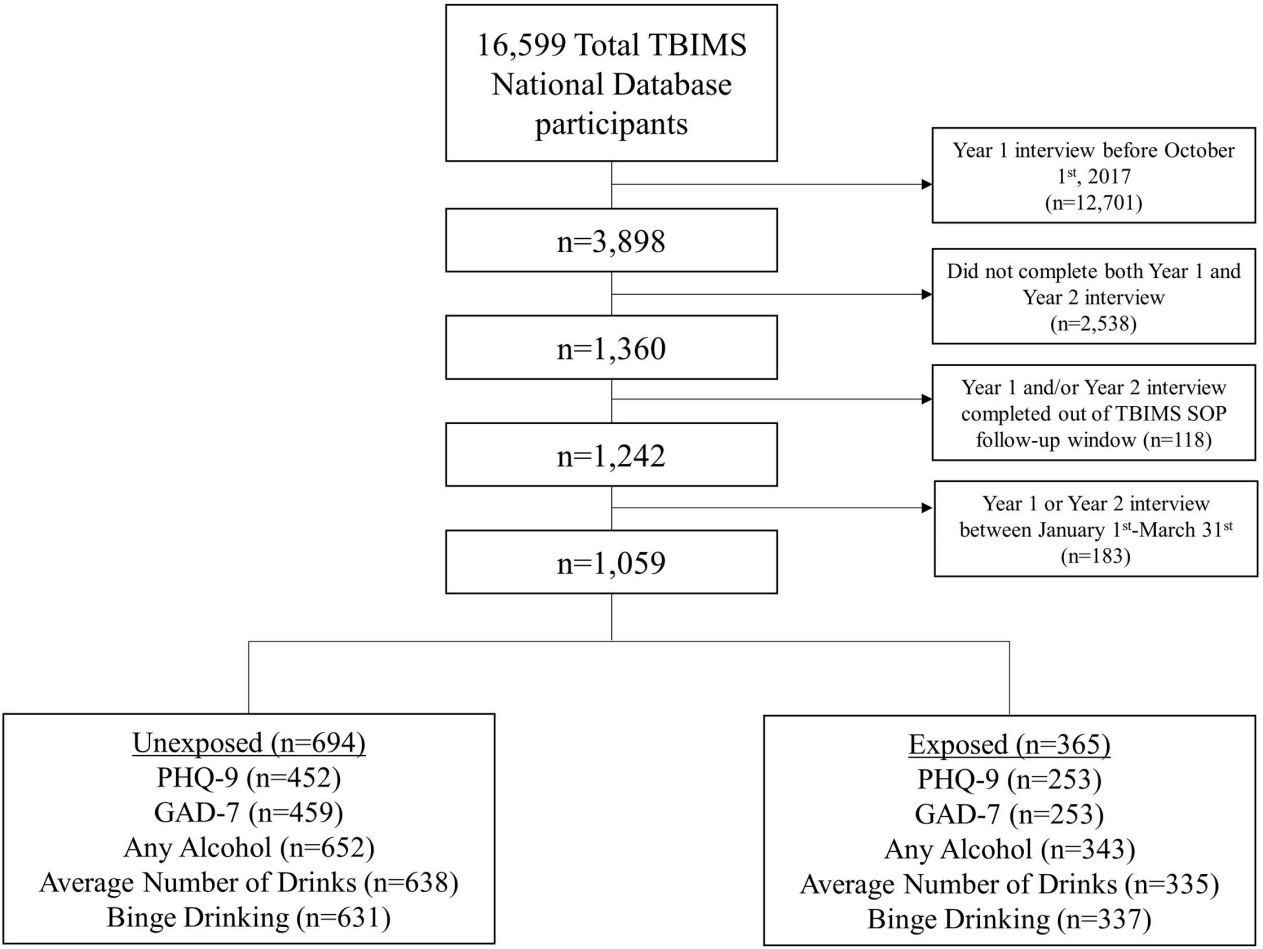
Study flow diagram. Derivation of analytic sample for alcohol use and mood variables.

For the current study, we selected participants, or their proxies, who had completed year 1 (Y1) and year 2 (Y2) post-injury follow-up interviews between October 2, 2017 and January 15, 2021. We defined our primary study exposure, “pandemic-exposed”, as participants with TBI who completed their Y1 post-injury interview before January 1, 2020 and their Y2 interview during the COVID-19 pandemic; and “unexposed” as those who completed both Y1 and Y2 interviews before January 1, 2020. We operationally defined the start of the pandemic as April 1, 2020, as this conservatively represented a point in time by which most regions of the U.S. were impacted by the pandemic. January 1, 2020-March 30, 2020 was considered an ambiguous period with regard to pandemic exposure; participants interviewed during this period were excluded for the purposes of our analyses. Thus, there were 1,059 participants with eligible follow-up interviews. Of these, n = 694 (66%) were pandemic-unexposed and n = 365 (34%) were pandemic-exposed (Fig 1 for the timeline of Y1 and Y2 interview dates for pandemic-unexposed and pandemic-exposed participants). We also provide a flow diagram showing the derivation of the analytic sample in Fig 2. The sample with PHQ-9 and GAD-7 was lower than the sample with alcohol use variables because mood measures could only be completed by participants with TBI, while alcohol use could be reported by either participant or proxy.

### Measures

#### Alcohol use

We determined alcohol use by asking participants (or their proxy) if they (the participant) had at least one drink in the month prior to the interview and the number of days per week or month these beverages were consumed. For those who reported drinking, they were asked how many drinks on average were ingested on days they drank. A ‘drink’ was operationally defined as one can or bottle of beer, glass of wine, can or bottle of wine cooler, cocktail, or shot of liquor. In accordance with the National Institute on Alcohol Abuse and Alcoholism (NIAAA) [30] definition of binge drinking, participants (or their proxy) were asked how many times during the past month they consumed 5+ drinks (males) or 4+ drinks (females) on one occasion. From these questions, we constructed the following outcomes: past month any drinking (y/n), average number of drinks per drinking occasion, and past month binge drinking (y/n).

#### Depression

We used the *Patient Health Questionnaire-9* (PHQ-9) to assess depressive symptom severity. We calculated a total score by summing each of the PHQ-9 items (range 0–27) with higher scores indicating greater depressive symptomatology. The criterion, construct, and external validity of the PHQ-9 have been well established using large samples from a range of patient populations including individuals with moderate-severe TBI [19, 31, 32].

#### Anxiety

We measured anxiety symptom severity using the *Generalized Anxiety Disorder 7*-*item scale* (GAD-7). The GAD-7 is a self-report screening questionnaire of generalized anxiety symptom severity [33]. We calculated a total score by summing each of the GAD-7 items (range 0–21) with higher scores indicating the presence of greater anxiety symptoms.

#### Covariates

We included the following sociodemographic covariates: age at injury, racial/ethnic identity, educational attainment, and primary rehabilitation payor source. We considered these injury-related characteristics: mechanism of injury, time to follow motor commands, time until emergence of post-traumatic amnesia, and pre-index TBI history (i.e., injuries prior to the incident TBI that qualified the individual for TBIMS participation). We included the following clinical characteristics: acute and rehabilitation lengths of stay, cranial surgery status, residential status after rehabilitation discharge, and Functional Independence Measure (FIM) scores at rehabilitation discharge.

### Statistical analysis

We used the quasi-experimental DiD design for this secondary analysis of the TBIMS National Database. DiD models are a well-established methodology used in public health, economics, and program evaluation [34–36]. This method compares longitudinal panel data between an exposed group and a counterfactual, unexposed group [36].

We constructed a series of DiD models using longitudinal generalized estimating equation (GEE) regression for all outcomes (alcohol use, depressive and anxiety symptoms), which facilitate estimation of population-average marginal effects while accounting for within-subject correlation of repeated observations from the same participant. Each model included a DiD coefficient, a follow-up period by pandemic exposure interaction representing differences in outcome scores over time modified by pandemic status. We adjusted GEE models for the covariates age, sex, race, and time to follow commands. For any alcohol use and binge drinking outcomes, we used GEE models with a binary distribution and logit link. For average number of drinks, we used GEE models with a negative binomial distribution with log link. For continuous outcomes of depressive symptoms (PHQ-9 total) and anxiety symptoms (GAD-7 total), we used GEE models with a Gaussian distribution and identity link. For all models, we used the *margins* and *marginsplot* commands in STATA 16.1 [37] to plot the predictions from the GEE model fit by pandemic status and follow-up period to illustrate the DiD trend for each outcome.

#### Subgroup analyses

We conducted pre-specified subgroup analyses by age (± 65 years old), sex, and race/ethnicity (White, Black, and Hispanic ethnicity). For Hispanic subgroup analyses, we included participants if they identified as Hispanic on the race/ethnicity question and/or a separate TBIMS question about Hispanic origin [38]. For each subgroup, we ran the same GEE models as primary analysis that included the follow-up period by pandemic exposure interaction, and adjusted for the same covariates (except those directly stratified on). For age-stratified models (± 65 years old), we controlled for chronological age to adjust for any residual confounding.

#### Checking assumptions and biases

There are fundamental assumptions underlying DiD models to facilitate causal interpretation [36]. Detailed explanation of our methods, which used historical TBIMS data from 2015–2016 to check the assumption of parallel trend, are provided in **S1 Methods.** Briefly, this assumption states that, in the absence of exposure, the exposed and unexposed groups would follow the same trajectory of outcome. Of note, the parallel trend assumption does not presuppose that exposure groups be balanced on outcome variables at baseline (e.g., Y1). We also compared those with Y1 data and missing Y2 data to the analytic sample to evaluate any potential selection biases due to attrition at Y2.

#### Sensitivity analyses

We conducted sensitivity analyses of the primary models for any alcohol use, average number of drinks, and binge drinking by excluding persons who were pre-injury alcohol abstainers to test whether conclusions were similar among a subsample of pre-injury drinkers.

## Results

### Sample characteristics by exposure status

The sample consisted of 1,059 participants with moderate-severe TBI (n = 694 pandemic-unexposed; n = 365 pandemic-exposed) (see **Table 1)**. The pandemic-exposed group was largely similar to the pandemic-unexposed group in demographic, injury, and clinical characteristics.

**Table 1:** Characteristics of sample by COVID-19 pandemic exposure.

	Pandemic Unexposed (n = 694)	Pandemic Exposed (n = 365)	p-value
**Demographic characteristics**			
Age at injury, Mean (SD)	45.5 (20.1)	47.3 (20.0)	0.157
Sex, Men (%)	519 (75.3%)	265 (72.6%)	0.335
Race, n (%)			0.293
White	442 (63.7%)	240 (65.9%)	
Black	120 (17.3%)	60 (16.5%)	
Hispanic	99 (14.3%)	40 (11.0%)	
Other	33 (4.8%)	24 (6.6%)	
Education, n (%)			0.353
Less than HS	144 (20.9%)	85 (23.4%)	
HS+	544 (79.1%)	278 (76.6%)	
Primary rehabilitation payor source, n (%)			0.487
	Private insurance	290 (42.2%)		144 (39.6%)
Medicare or Medicaid	235 (34.2%)	138 (37.9%)	
Other	162 (23.6%)	82 (22.5%)	
**Injury characteristics**			
Mechanism of injury, n (%)			0.825
Motor vehicle	254 (37.0%)	137 (37.6%)	
Fall	247 (36.0%)	138 (37.9%)	
Any violence	50 (7.3%)	24 (6.6%)	
Other	136 (19.8%)	65 (17.9%)	
GCS score, Median (IQR)	13 (6–15)	13 (7–15)	0.487
TFC (days), Median (IQR)	1 (0.5–8)	1 (0.5–5)	0.008*
Duration of PTA (days), Median (IQR)	19 (4–36)	16 (4–34)	0.317
Pre-index lifetime history of TBI, n (%)	174 (25.3%)	81 (22.4%)	0.295
**Clinical characteristics**			
Acute hospital length of stay, Mean (SD)	19.8 (15.5)	20.5 (20.0)	0.507
Inpatient rehabilitation length of stay, Mean (SD)	25.3 (27.3)	23.7 (23.7)	0.193
Craniotomy or craniectomy, n (%)	174 (25.3%)	95 (26.0%)	0.794
FIM Motor at Rehabilitation discharge, Mean (SD)	65.4 (18.0)	65.2 (17.5)	0.696
FIM Cognitive at Rehabilitation Discharge, Mean (SD)	23.7 (6.5)	23.8 (6.8)	0.645
Residence after inpatient			0.279
rehabilitation discharge, n (%)			
Private residence	551 (80.2%)	278 (76.2%)	
Nursing home/adult home	13 (1.9%)	10 (2.7%)	
Other	123 (17.9%)	77 (21.1%)	

Abbreviations: Glasgow Coma Scale, GCS; Time to Follow Commands, TFC, Post-traumatic amnesia, PTA; Functional Independence Measure, FIM.

*statistically significant at α = 0.05.

### Evaluating model assumptions and potential biases

Historical TBIMS data provided us with reasonable confidence that parallel trend assumptions were satisfied for all outcomes in our primary sample and all tested subgroups (**S1–S17 Figs)**.

Those lost to follow-up were slightly older, more often had public insurance, more likely injured in a fall, were less likely to have a pre-index TBI history, and had lower FIM Cognitive scores at inpatient rehabilitation discharge than those followed. However, those lost to follow-up did not significantly differ from the analytic sample on any alcohol use or mood variables at Y1 post-injury (**S1 Table**).

### Difference-in-difference models: Primary analysis

**Fig 3:**
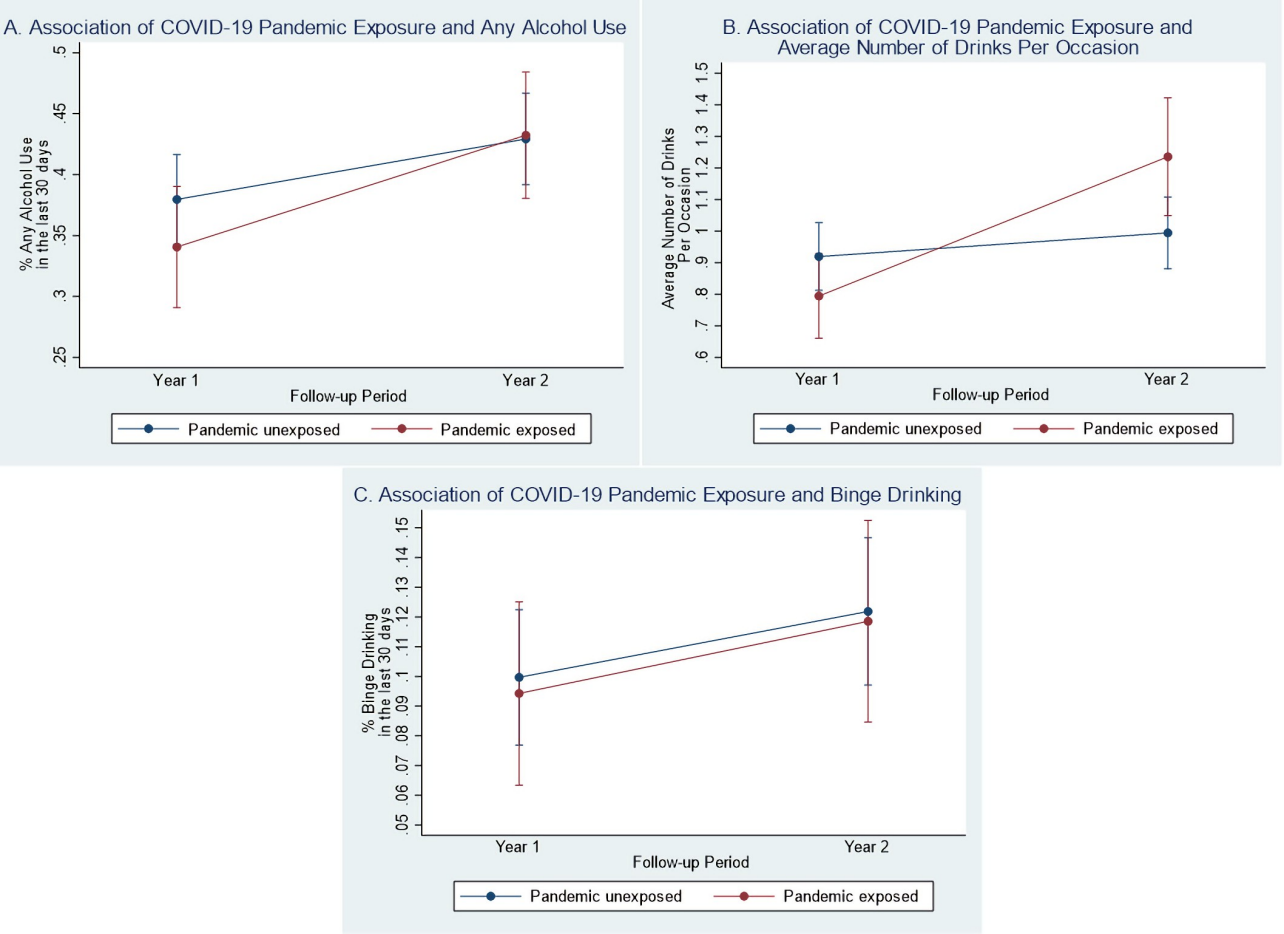
Difference-in-difference plots of year 1 and year 2 mood and alcohol use by COVID-19 pandemic status (primary analysis). (A) Y1 and Y2 model-fitted values for probability of any alcohol use in the last 30 days by pandemic status (interaction p-value = 0.167). (B) Y1 and Y2 model-fitted values for average number of drinks per occasion by pandemic status (interaction p-value = 0.001). (C) Y1 and Y2 model-fitted values for probability of any binge drinking in the last 30 days by pandemic status (interaction p-value = 0.903). (D) Y1 and Y2 model-fitted values for PHQ-9 total score by pandemic status (interaction p-value = 0.930). (E) Y1 and Y2 model-fitted values for GAD-7 total score by pandemic status (interaction p-value = 0.158).

**Fig 4:**
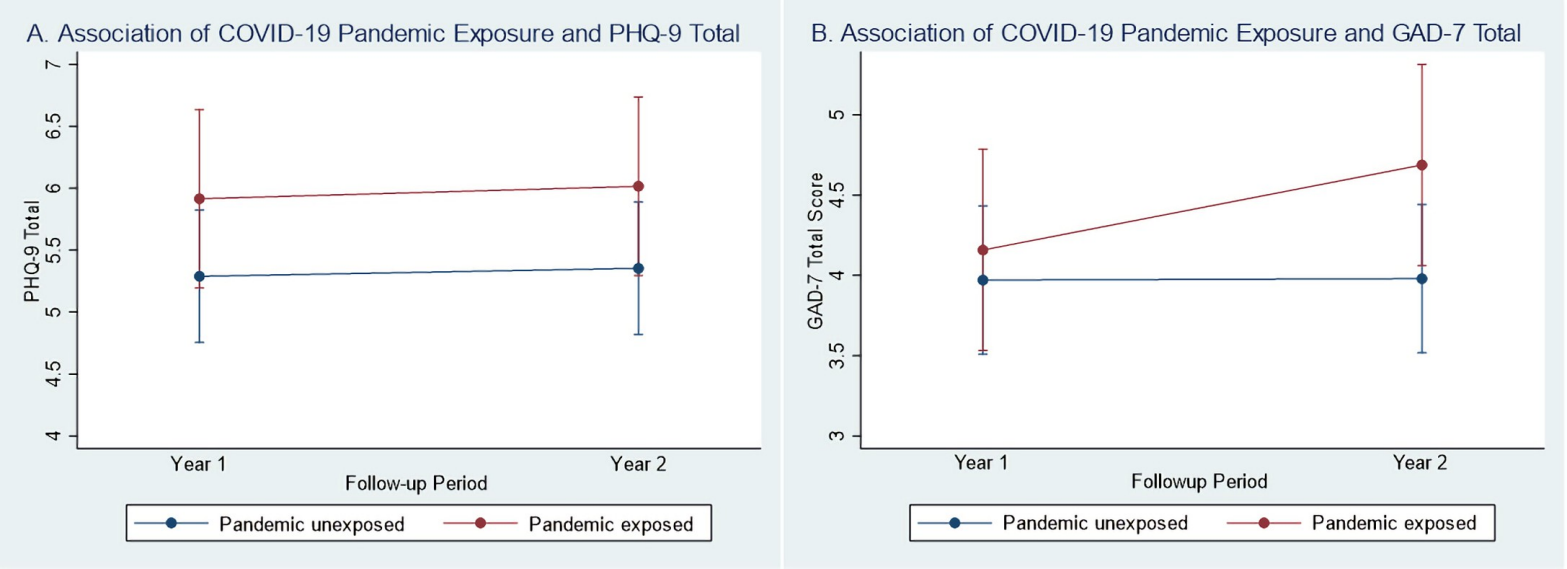
Difference-in-difference plots of year 1 and year 2 mood by COVID-19 pandemic status (primary analysis). (A) Y1 and Y2 model-fitted values for PHQ-9 total score by pandemic status (interaction p-value = 0.930). (B) Y1 and Y2 model-fitted values for GAD-7 total score by pandemic status (interaction p-value = 0.158).

For alcohol use, pandemic-exposed individuals with TBI reported greater increases in their average quantity of drinks per occasion from Y1 to Y2 post-injury compared to the pandemic-unexposed group (β = 0.36, 95% CI: 0.16, 0.57, p = 0.001; see **Table 2**). There was insufficient evidence that probability of any alcohol use in the last month (β = 0.19, 95% CI: -0.98, 0.46, p = 0.167) and binge drinking in the last month (β = 0.03, 95% CI: -0.28, 0.54, p = 0.903) varied over time between exposure groups (see **Fig 3A–3C**). There was insufficient evidence of a difference in depressive symptoms (β = 0.04, 95% CI: -0.76, 0.84, p = 0.930) and anxiety symptoms (β = 0.52, 95% CI: -0.20, 1.25, p = 0.158) over time by pandemic exposure (see **Fig 4A and 4B**).

**Table 2:** Difference-in difference analysis of alcohol use and mood by COVID-19 pandemic exposure status.

Outcome					
Any alcohol use in the last month	COVID-19 pandemic exposure	Follow-up period	Ncases^¥^ (%)	DiD Parameter Estimate^§^ (95% CI)	P-value
	No (n = 652)	Year 1	245 (37.6%)	0.19 (-0.08, 0.46)	0.167
		Year 2	277 (42.5%)		
	Yes (n = 343)	Year 1	118 (34.4%)		
		Year 2	149 (43.4%)		
Average number of drinks per occasion	COVID-19 pandemic exposure	Follow-up period	Mean^¥^ (SE)	DiD Parameter Estimate^€^ (95% CI)	P-value
	No (n = 638)	Year 1	0.91 (1.83)	0.36 (0.16, 0.57)	0.001*
		Year 2	1.00 (1.72)		
	Yes (n = 335)	Year 1	0.78 (1.42)		
		Year 2	1.23 (2.11)		
Any binge drinking in the last month	COVID-19 pandemic exposure	Follow-up period	Ncases^¥^ (%)	DiD Parameter Estimate^§^ (95% CI)	P-value
	No (n = 631)	Year 1	63 (10.0%)	0.03 (-0.48, 0.54)	0.903
		Year 2	77 (12.2%)		
	Yes (n = 337)	Year 1	33 (9.8%)		
		Year 2	41 (12.2%)		
PHQ-9	COVID-19 pandemic exposure	Follow-up period	Mean^¥^ (SD)	DiD Parameter Estimate^┼^ (95% CI)	P-value
	No (n = 452)	Year 1	5.34 (5.97)	0.04 (-0.76, 0.84)	0.930
		Year 2	5.39 (5.63)		
	Yes (n = 253)	Year 1	5.90 (6.04)		
		Year 2	6.06 (6.04)		
GAD-7	COVID-19 pandemic exposure	Follow-up period	Mean^¥^ (SE)	DiD Parameter Estimate^┼^ (95% CI)	P-value
	No (n = 459)	Year 1	4.00 (5.37)	0.52 (-0.20, 1.25)	0.158
		Year 2	4.00 (5.08)		
	Yes (n = 253)	Year 1	4.14 (5.06)		
		Year 2	4.70 (5.13)		

Abbreviations: Patient Health Questionnaire-9, PHQ-9; Generalized Anxiety Disorder-7, GAD-7; Difference-in- Difference, DiD

^¥^Descriptive measure, not model-based or adjusted for covariates

^┼^Estimate represents pandemic exposure*followup period interaction parameter estimate from GEE Model with Gaussian distribution and identity link. The GEE model adjusted for age at injury, sex, race, and time to follow commands in days (interpreted as DiD in PHQ-9/GAD-7 between pandemic exposed vs. unexposed from year 1 to year 2).

^§^Estimate represents pandemic exposure*followup period interaction parameter estimate from GEE Model with binomial distribution and logit link. The GEE model adjusted for age at injury, sex, race, and time to follow commands in days (interpreted as DiD in any alcohol use/any binge drinking between pandemic exposed vs. unexposed from year 1 to year 2).

^€^Estimate represents pandemic exposure*followup period interaction parameter estimate from GEE Model with negative binomial distribution and log link. The GEE model adjusted for age at injury, sex, race, and time to follow commands in days (interpreted as DiD in average number of drinks consumed per occasion between pandemic exposed vs. unexposed from year 1 to year 2).

### Subgroup analyses among age, sex, and race/ethnicity subgroups

**Fig 5:**
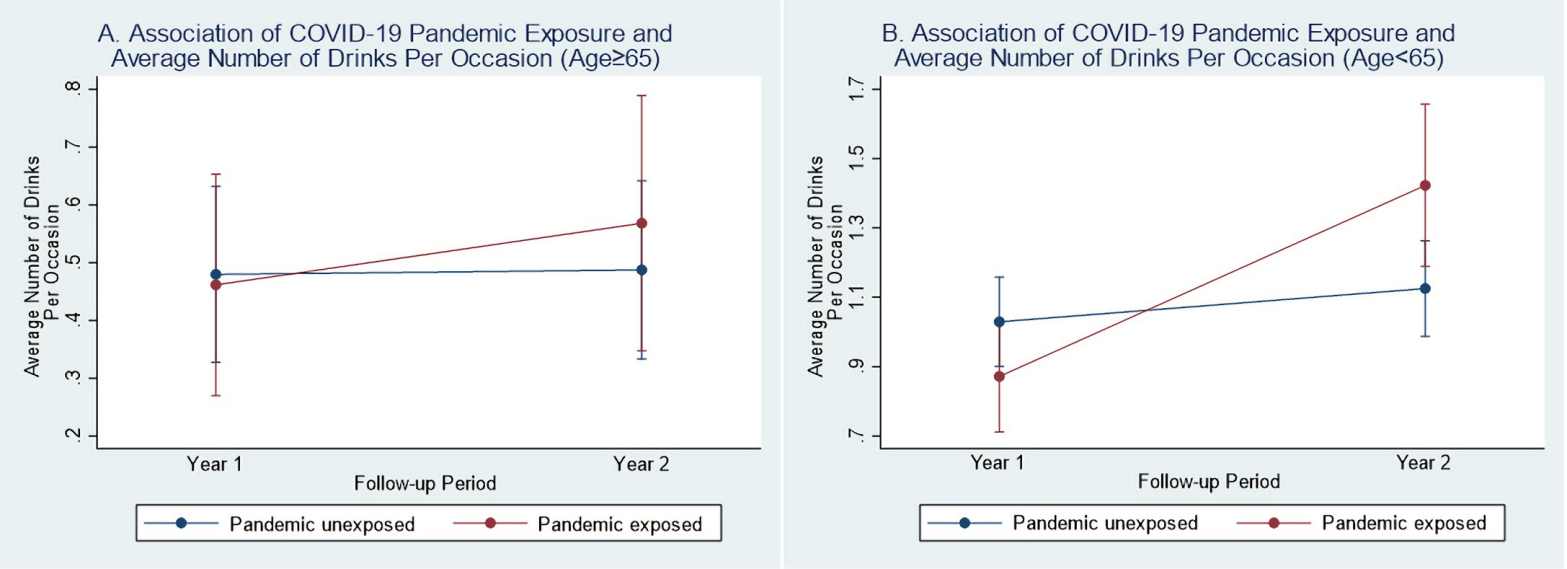
Difference-in-difference plots of year 1 and year 2 average number of drink by COVID-19 pandemic status (among age subgroups). (A) Y1 and Y2 model-fitted values for average number of drinks per occasion by pandemic status among adults age ≥65 (interaction p-value = 0.474). (B) Y1 and Y2 model-fitted values for average number of drinks per occasion by pandemic status among adults age <65 (interaction p-value = 0.001).

**Fig 6:**
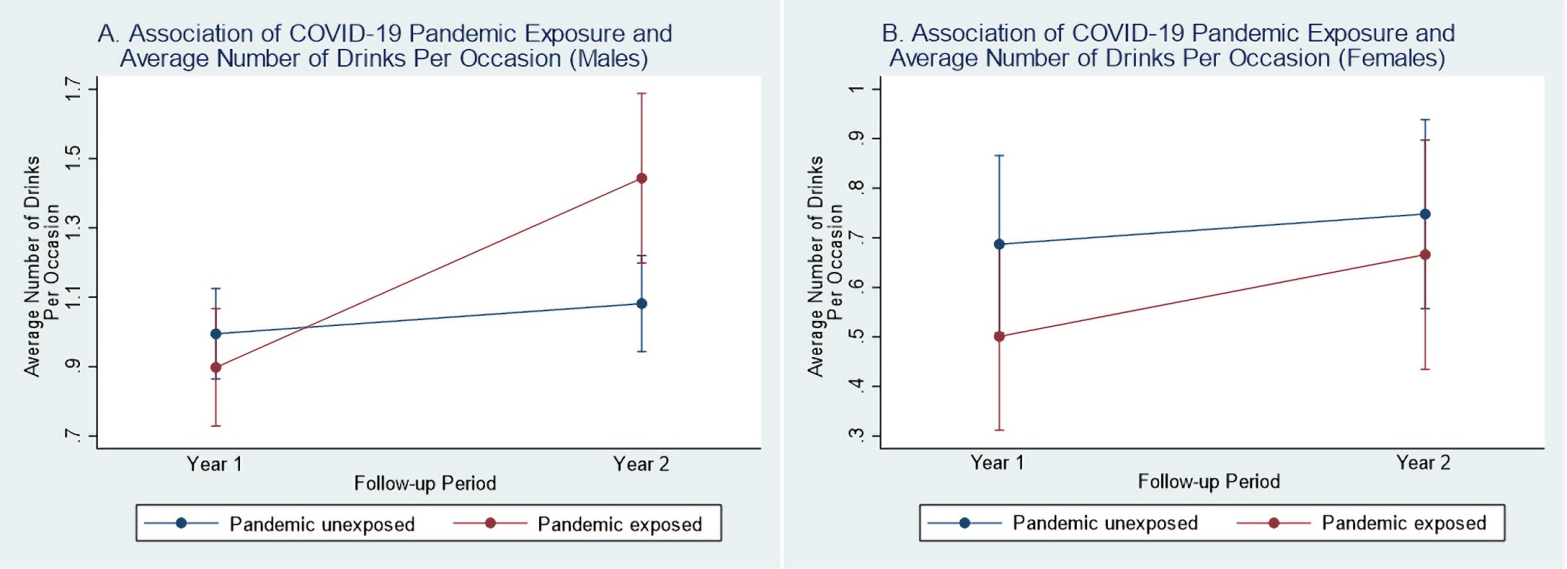
Difference-in-difference plots of year 1 and year 2 average number of drink by COVID-19 pandemic status (among sex subgroups). (A) Y1 and Y2 model-fitted values for average number of drinks per occasion by pandemic status among males (interaction p-value = 0.001). (B) Y1 and Y2 model-fitted values for average number of drinks per occasion by pandemic status among females (interaction p-value = 0.401).

Pandemic-exposed persons who were less than 65 years old (β = 0.40, 95% CI: 0.17, 0.63, p = 0.001) and male (β = 0.39, 95% CI: 0.16, 0.62, p = 0.001) had greater increases in their average number of drinks per occasion from Y1 to Y2 post-injury compared to their pandemic-unexposed demographic counterparts (see **Figs 5A and 5B and 6A and 6B and S2 Table**).

**Fig 7:**
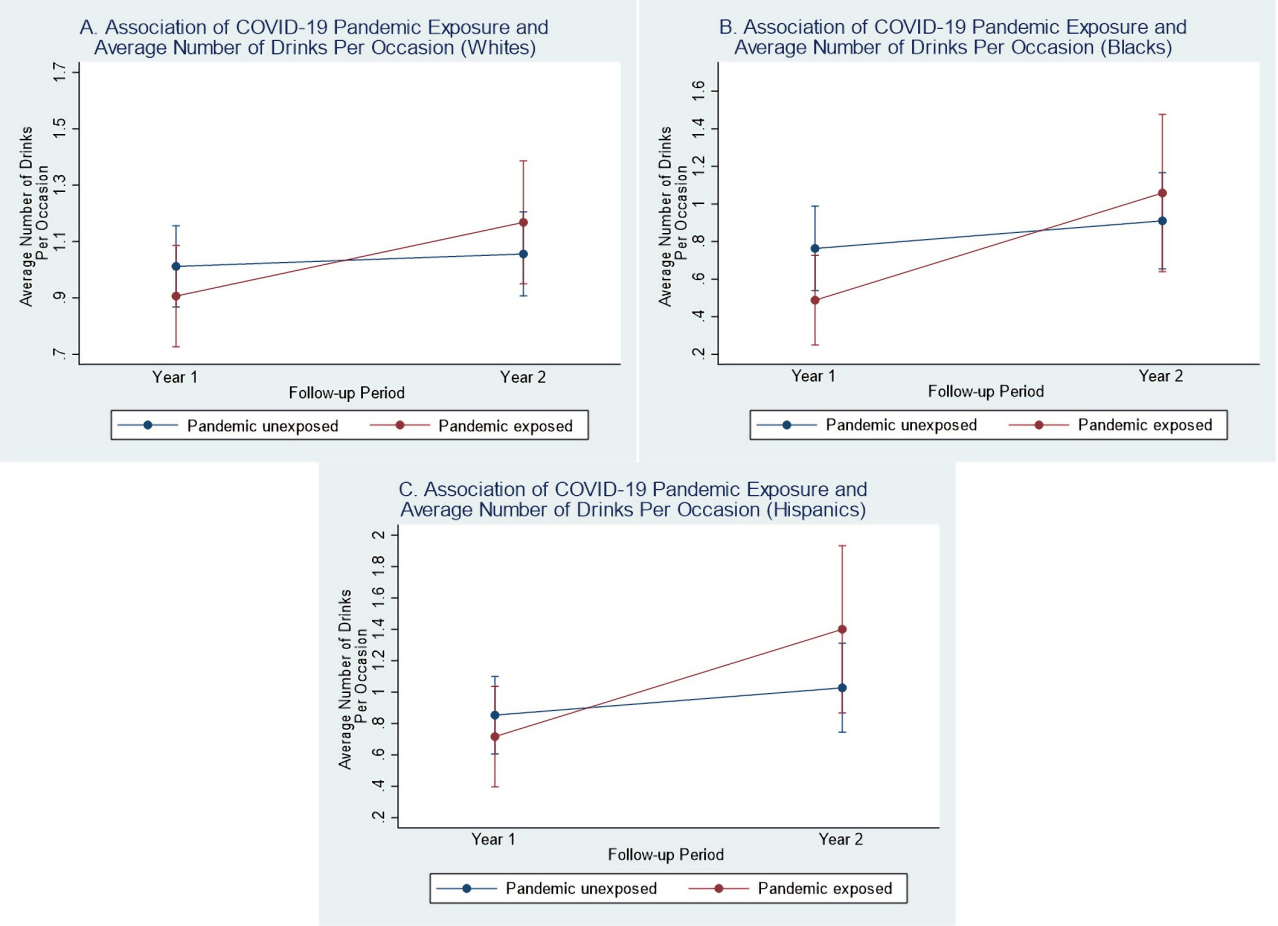
Difference-in-difference plots of year 1 and year 2 average number of drink by COVID-19 pandemic status (among race subgroups). (A) Y1 and Y2 model-fitted values for average number of drinks per occasion by pandemic status among Whites (interaction p-value = 0.089). (B) Y1 and Y2 model-fitted values for average number of drinks per occasion by pandemic status among Blacks (interaction p-value = 0.046). (C) Y1 and Y2 model-fitted values for average number of drinks per occasion by pandemic status among Hispanics (interaction p-value = 0.045).

Black (β = 0.60, 95% CI: 0.01, 1.19, p = 0.046) and Hispanic (β = 0.48, 95% CI: 0.01, 0.96, p = 0.045) pandemic-exposed participants had greater increases in their average number of drinks from Y1 to Y2 compared to their pandemic-unexposed counterparts (see **Fig 7A–7C**). There was insufficient evidence of change in probability of any alcohol use or past month binge drinking by pandemic status among any demographic subgroups (**S3–S4 Tables**).

For depressive symptoms, there was insufficient evidence that the DiD parameters were significant among any age, sex, or race/ethnicity subgroups (**S5 Table**). For anxiety symptoms, among Hispanics, persons who were exposed to the pandemic had on average a 2.4-point greater increase in their GAD-7 total scores from Y1 to Y2 compared to changes over time among Hispanics unexposed to the pandemic (β = 2.35, 95% CI: 0.25, 4.47, p = 0.028). No other demographic subgroup had statistically significant differences in their anxiety scores over time by exposure group (**S6 Table**).

### Sensitivity analyses

Sensitivity analyses in which we excluded persons who were pre-injury alcohol abstainers showed findings largely similar to the primary alcohol use analyses (**S7 Table**).

## Discussion

Given that individuals with disabilities have been found to be differentially impacted by the COVID-19 pandemic [12–14], individuals with moderate-to-severe TBI may be susceptible to direct and/or indirect effects of the COVID-19 pandemic. However, few studies to date have investigated how the pandemic has affected persons with TBI. The current study used a novel quasi-experimental DiD design to evaluate how alcohol use and mood among individuals with TBI has changed as a result of the pandemic, including an evaluation of the pandemic’s impact on demographic subgroups.

Our data indicated that the average number of drinks per occasion increased more from Y1 to Y2 post-injury among pandemic-exposed persons with TBI compared to those unexposed. The largest increases were seen among males, persons under 65 years old, and Black or Hispanic racial/ethnic minorities. We did not find evidence of differences in the rate of engaging in any alcohol use or past month binge drinking from Y1 to Y2 by pandemic exposure. This is consistent with research [39] from the early months of the pandemic which found that adults consumed more drinks per day in April 2020 compared to February 2020; yet, unlike this prior study, we did not observe increases in binge drinking during the pandemic for persons with moderate-to-severe TBI.

Our study findings suggest factors associated with the pandemic and the context in which people were drinking may have facilitated increases in the quantity of drinks consumed during a typical drinking occasion. We speculate these changes reflect a convergence of factors such as social distancing measures during the pandemic that resulted in more drinking at home without worrying about driving home safely, purchasing alcohol in larger quantities due to availability concerns or to reduce shopping trips during the pandemic, or relaxing of state alcohol purchasing policies during the pandemic (e.g., increases in alcohol delivery options, restaurants being allowed to provide take-home alcohol with orders) [40]. While beyond the data that we had available in this study, it is likely some individuals had increases in occasions of solitary drinking during the pandemic due to the stay-at-home orders and most social activities being cancelled. Solitary drinking has unique risks for experiencing alcohol consequences or developing an alcohol use disorder [41, 42].

The psychological and social impact of living through the pandemic has been evident in studies in the general population [8, 43–47], and recent work has shown that one-third of individuals with TBI have identified mental health challenges and social isolation as key barriers to effective coping with the COVID-19 pandemic [16]. The Household Pulse Survey [48] found that anxiety has been particularly high among Hispanic adults and minority racial groups, and also for persons with a disability [48]. Data from the Centers for Disease Control and Prevention revealed that Hispanic adults reported more psychosocial distress than non-Hispanic adults during the pandemic due to instability in housing and food, and death of a loved one [45]. Similarly, in our study, we found that the pandemic resulted in increasing anxiety symptoms particularly among Hispanic persons with TBI. Though we did not observe population-level differences in mood by pandemic exposure in our study, mental health should still be continually monitored in TBI populations moving forward. Individuals with moderate-to-severe TBI already struggle with anxiety [49], loneliness [50], and limited social participation compared to non-injured peers [51, 52]. These daily challenges would likely only worsen during a global pandemic due to fear of contracting the virus and recommendations or mandates to limit in-person social interaction. Among the reasons for decreased socialization are difficulty navigating masked social interactions [16] and barriers to the use of on-line technology platforms [53, 54] for socialization. These new challenges, superimposed on the already existing challenges in this area faced by individuals with TBI, would only add to the mental health challenges characteristic of the COVID-19 pandemic.

The pattern of subgroup findings reported here––specifically, that the largest increases in average drinks per occasion were among Black or Hispanic racial/ethnic minorities, together with substantial increases in anxiety symptoms among Hispanic individuals exposed to the pandemic––may suggest that factors other than the pandemic played a role in the differences observed. Previous research has found Black/non-Hispanic adults and Hispanic women have had the largest increases in alcohol consumption during the pandemic, consuming more drinks per day than White, non-Hispanic adults, with the exception of women with children under 5 years old [39]. And alcohol consumption is inextricably linked with anxiety [55]. The political and sociocultural climate in the U.S. during the pandemic can be characterized by political discord and heightened racial tensions, and it will be impossible to disentangle the relative contributions of multiple stressors on the results observed herein. However, the observation that racial and ethnic minorities with TBI who were exposed to the pandemic experienced greater increases in alcohol use and anxiety may reflect disproportionately detrimental ambient stress and related consequences during the time period studied.

The TBIMS offers a unique opportunity to understand the impact of the pandemic on individuals with TBI because we have data on the same participants before and after pandemic and are able to compare this change to an unexposed comparison group with harmonized measures. However, there are some limitations in both the data available and our analysis approach. Though our binge drinking measure was consistent with NIAAA guidelines, we did not have information available in this study on alcohol use disorder nor, more generally, consequences of drinking. We were unable to determine whether participants had COVID-19, and did not have measures on the mediating psychosocial factors (e.g., loss of a loved one, discrimination) that may explain our results. The DiD approach requires non-missing data from Y1 and Y2, so it is possible that the most depressed/anxious individuals were not captured at Y2 due to higher attrition; however, this limitation is ameliorated by there being no significant differences in Y1 alcohol use and depression/anxiety between the analytic sample and those without Y2 data. Finally, consistent with other studies [27, 28, 45, 48], findings from our study highlight how systemic factors (e.g., access to healthcare and technology) affect individuals with TBI and from racial and ethnic minority groups. Though we did not have data available to measure these factors directly, this is an important area for future study.

## Conclusions

The current findings indicating an increase in the number of drinks consumed on a typical drinking occasion and anxiety suggest we should monitor alcohol consumption and mental health among individuals with TBI as the pandemic unfolds. In particular, adherence to low-risk drinking guidelines [56] for individuals with TBI can help mitigate future risk for substance use disorders [57].

## Supporting information

S1 Methods Parallel trend assumptions of difference-in-difference models. (DOCX)

S1 Fig Investigation of parallel trend historical analysis of alcohol use (overall sample). (TIF)

S2 Fig Investigation of parallel trend historical analysis depression and anxiety (overall sample). (TIF)

S3 Fig Investigation of parallel trend historical analysis of any alcohol (age subgroups). (TIF)

S4 Fig Investigation of parallel trend historical analysis of any alcohol (sex subgroups). (TIF)

S5 Fig Investigation of parallel trend historical analysis of any alcohol (race/ethnicity subgroups). (TIF)

S6 Fig Investigation of parallel trend historical analysis of average number of drinks per occasion (age subgroups). (TIF)

S7 Fig Investigation of parallel trend historical analysis of average number of drinks per occasion (sex subgroups). (TIF)

S8 Fig Investigation of parallel trend historical analysis of average number of drinks per occasion (race/ethnicity subgroups). (TIF)

S9 Fig Investigation of parallel trend historical analysis of binge drinking (age subgroups). (TIF)

S10 Fig Investigation of parallel trend historical analysis of binge drinking (sex subgroups). (TIF)

S11 Fig Investigation of parallel trend historical analysis of binge drinking (race/ethnicity subgroups). (TIF)

S12 Fig Investigation of parallel trend historical analysis of depression (age subgroups). (TIF)

S13 Fig Investigation of parallel trend historical analysis of depression (sex subgroups). (TIF)

S14 Fig Investigation of parallel trend historical analysis of depression (race/ethnicity subgroups). (TIF)

S15 Fig Investigation of parallel trend historical analysis of anxiety (age subgroups). (TIF)

S16 Fig Investigation of parallel trend historical analysis of anxiety (sex subgroups). (TIF)

S17 Fig Investigation of parallel trend historical analysis of anxiety (race/ethnicity subgroups). (TIF)

S1 Table Investigation of potential selection bias for completed year 2 follow-up after completed year 1 follow-up. (DOCX)

S2 Table Subgroup difference-in-difference analyses of average number of drinks by pandemic exposure status. (DOCX)

S3 Table Subgroup difference-in-difference analyses of any alcohol use in the last month by pandemic exposure status. (DOCX)

S4 Table Subgroup difference-in-difference analyses of binge drinking in the last month by pandemic exposure status. (DOCX)

S5 Table Subgroup difference-in-difference analyses of phq-9 by pandemic exposure status. (DOCX)

S6 Table Subgroup difference-in-difference analyses of gad-7 by pandemic exposure status. (DOCX)

S7 Table Sensitivity analysis of alcohol use outcomes excluding pre-injury abstainers. (DOCX)
