# Supplementary material for: Changes in alcohol use and mood during the COVID-19 pandemic among individuals with traumatic brain injury: A difference-in-difference study
Source: PLoS One. 2022 Apr 7;17(4):e0266422. doi: 10.1371/journal.pone.0266422 (PMC8989351; doi:10.1371/journal.pone.0266422)
Supplement: S1 Methods — (DOCX) [file pone.0266422.s001.docx]

S1 Methods:

*Parallel trend assumptions of difference-in-difference models*

Difference-in-difference models rely on the fundamental assumption of a “hypothetical” parallel trend in outcome scores over time between groups. This means that, in a counterfactual scenario of no exposure, the exposed and unexposed groups would follow the same trajectory of outcome. This assumption must be satisfied for each difference-in-difference model (overall sample and subgroups) in order to make casual inferences. To test this assumption in our study, we used historical data from the TBI Model Systems National Data from year 1 post-injury interview in 2015 and 2016, and compared to our unexposed group in the analytic sample (whose year 1 interviews occurred between 2017-2019). The historical comparison groups were mutually-exclusive from the unexposed analytic group. Furthermore, we compared the slope observed in the historical groups versus the unexposed analytic group to evaluate the parallel trend assumption in the primary analytic sample, and in all tested subgroups. The rationale was, if the unexposed analytic group had a similar slope of outcome compared to historical comparisons, then we are reasonably confident that this trend would have been observed in our exposed group. Of note, because the depression and anxiety scales were modified in the TBIMS beginning in 2017, we looked for graphical evidence of parallel trends between depression and anxiety scales between the historical comparison and the unexposed analytic group. For alcohol use variables, we further statistically compared trajectories between the historical comparison groups and the analytic unexposed group. To check assumptions of the binge drinking variable, we used the criteria of 5 or more drinks per occasion for both males and females, as the female-specific variable (4+ drinks) was only available beginning in 2017; therefore, was not available in historical cohort.

*Additional causal inference assumptions*

Notably, other causal inference assumptions were also met, including: allocation of exposure was not determined by study outcomes, no spillover effects of exposure (e.g., exposed and unexposed groups were mutually exclusive), and exchangeability between exposed and unexposed groups (e.g., exposure groups were otherwise similar besides their pandemic exposure).
