# Supplementary figures and images for: Changes in alcohol use and mood during the COVID-19 pandemic among individuals with traumatic brain injury: A difference-in-difference study
Source: PLoS One. 2022 Apr 7;17(4):e0266422. doi: 10.1371/journal.pone.0266422 (PMC8989351; doi:10.1371/journal.pone.0266422)

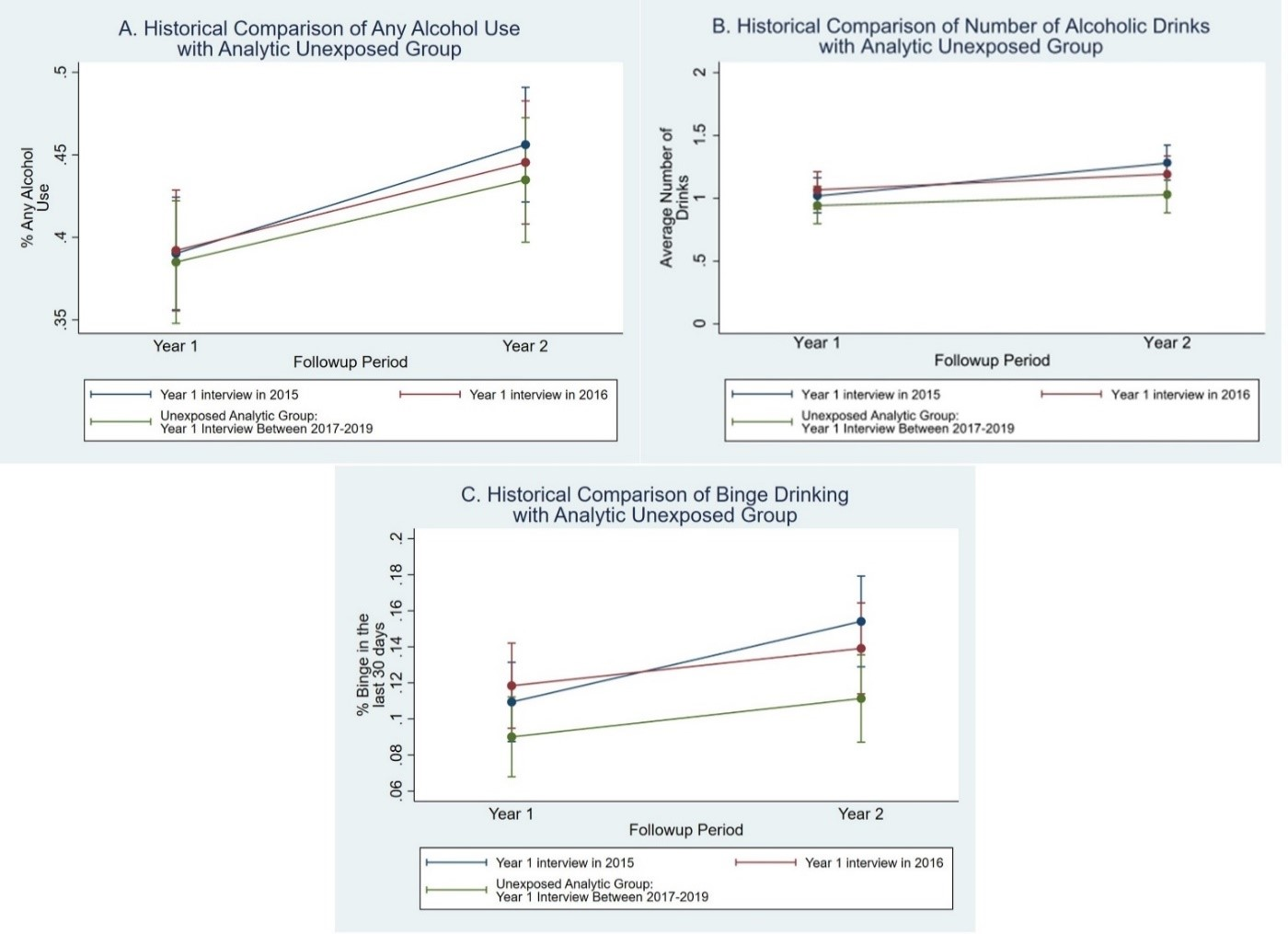

Supplement: S1 Fig — (TIF) [file pone.0266422.s002.tif]

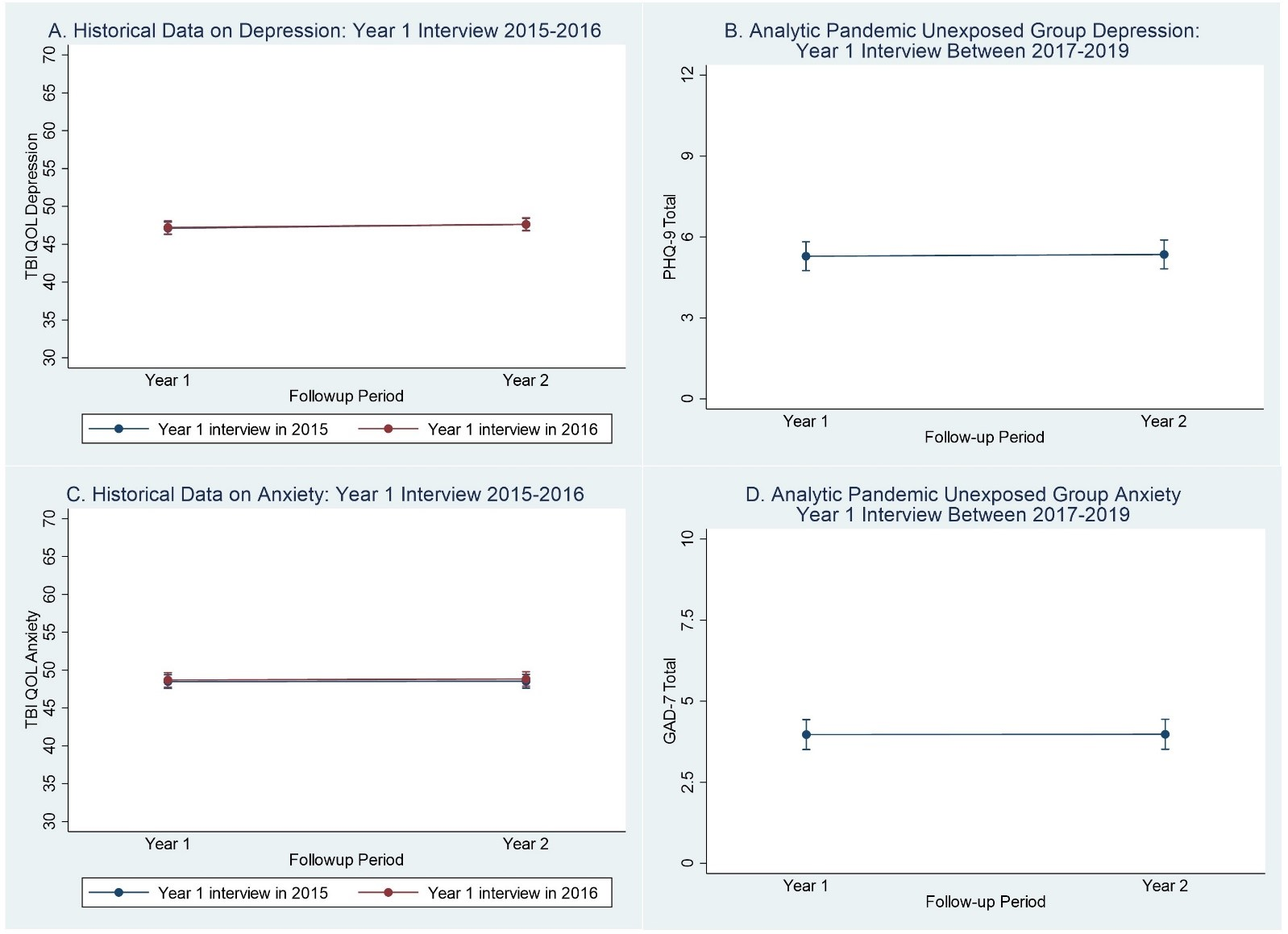

Supplement: S2 Fig — (TIF) [file pone.0266422.s003.tif]

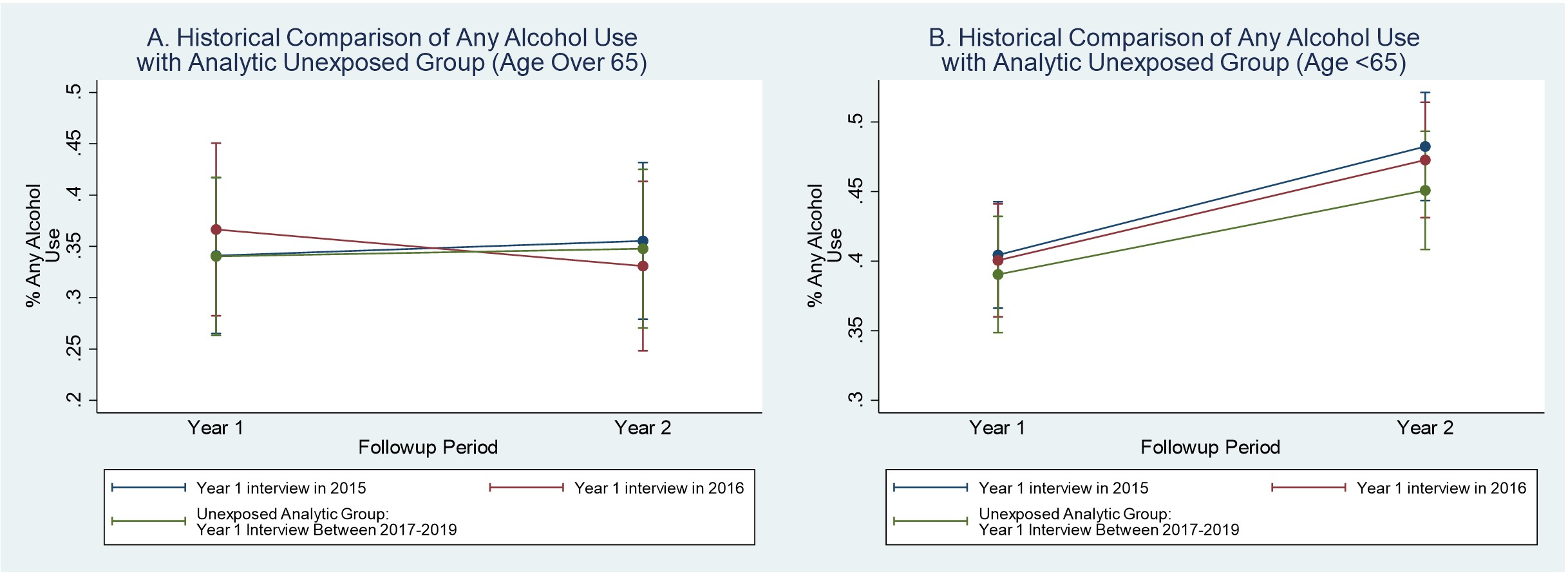

Supplement: S3 Fig — (TIF) [file pone.0266422.s004.tif]

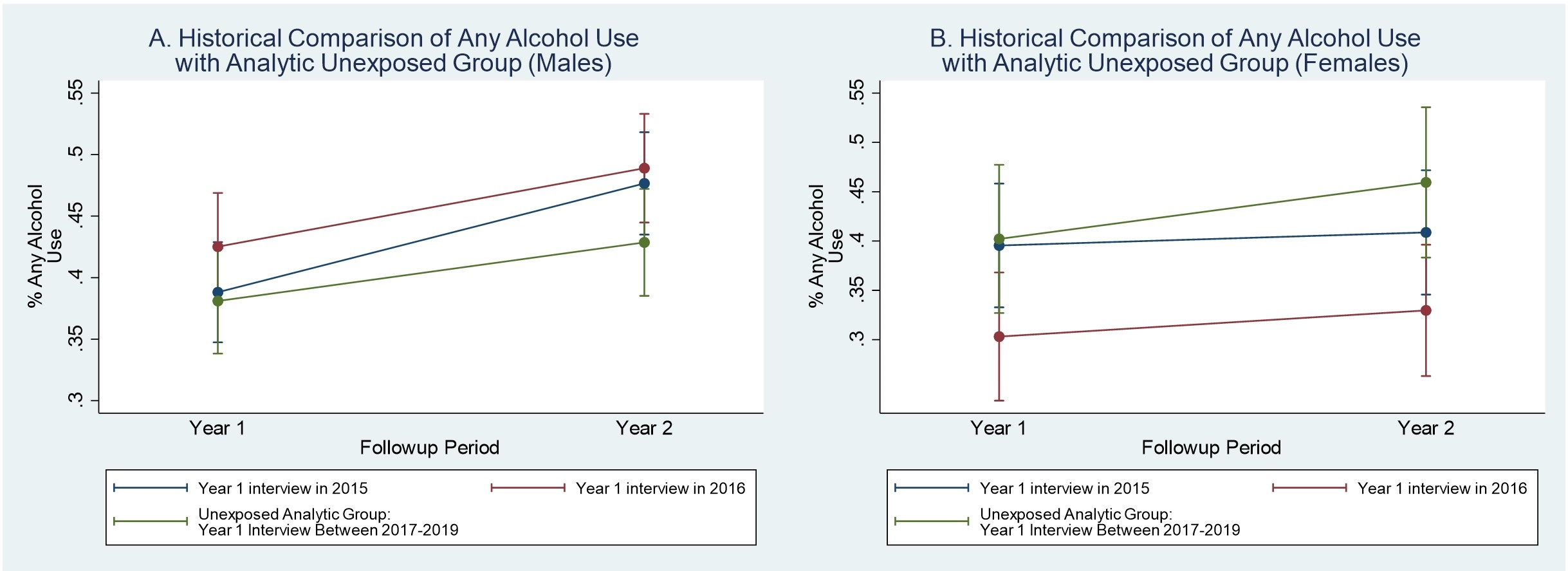

Supplement: S4 Fig — (TIF) [file pone.0266422.s005.tif]

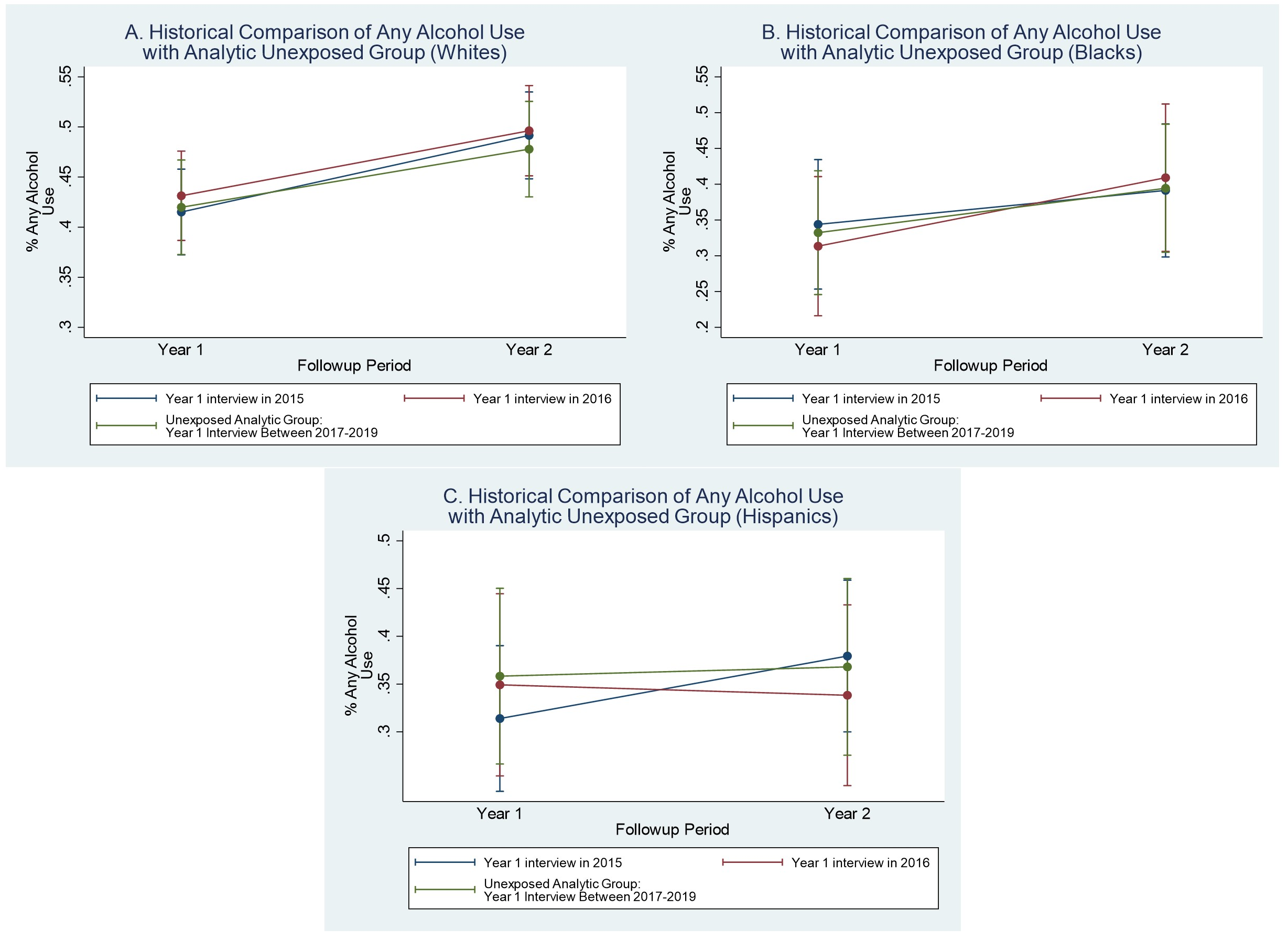

Supplement: S5 Fig — (TIF) [file pone.0266422.s006.tif]

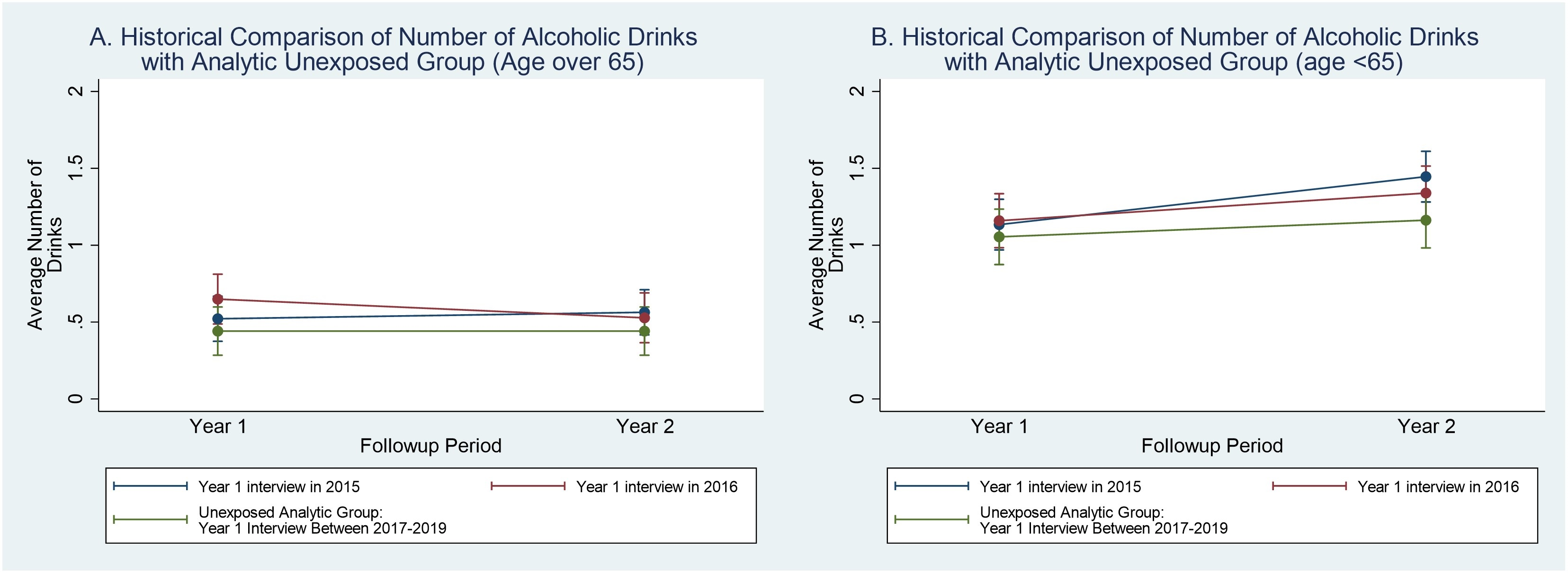

Supplement: S6 Fig — (TIF) [file pone.0266422.s007.tif]

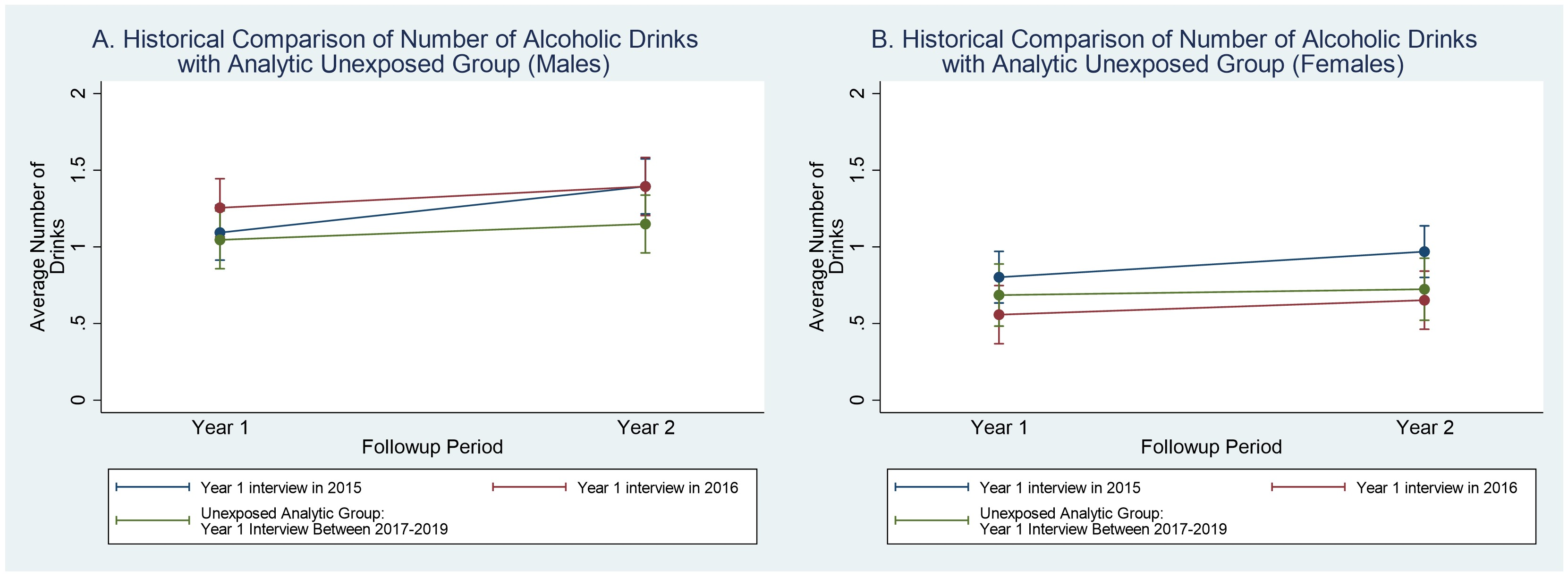

Supplement: S7 Fig — (TIF) [file pone.0266422.s008.tif]

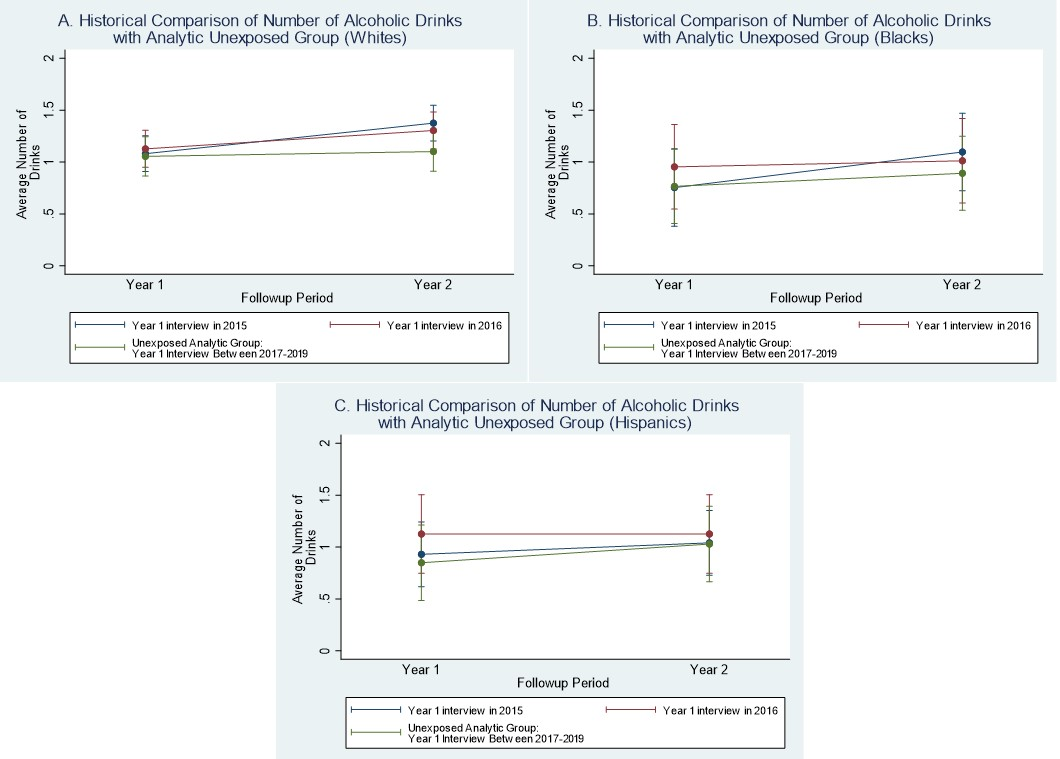

Supplement: S8 Fig — (TIF) [file pone.0266422.s009.tif]

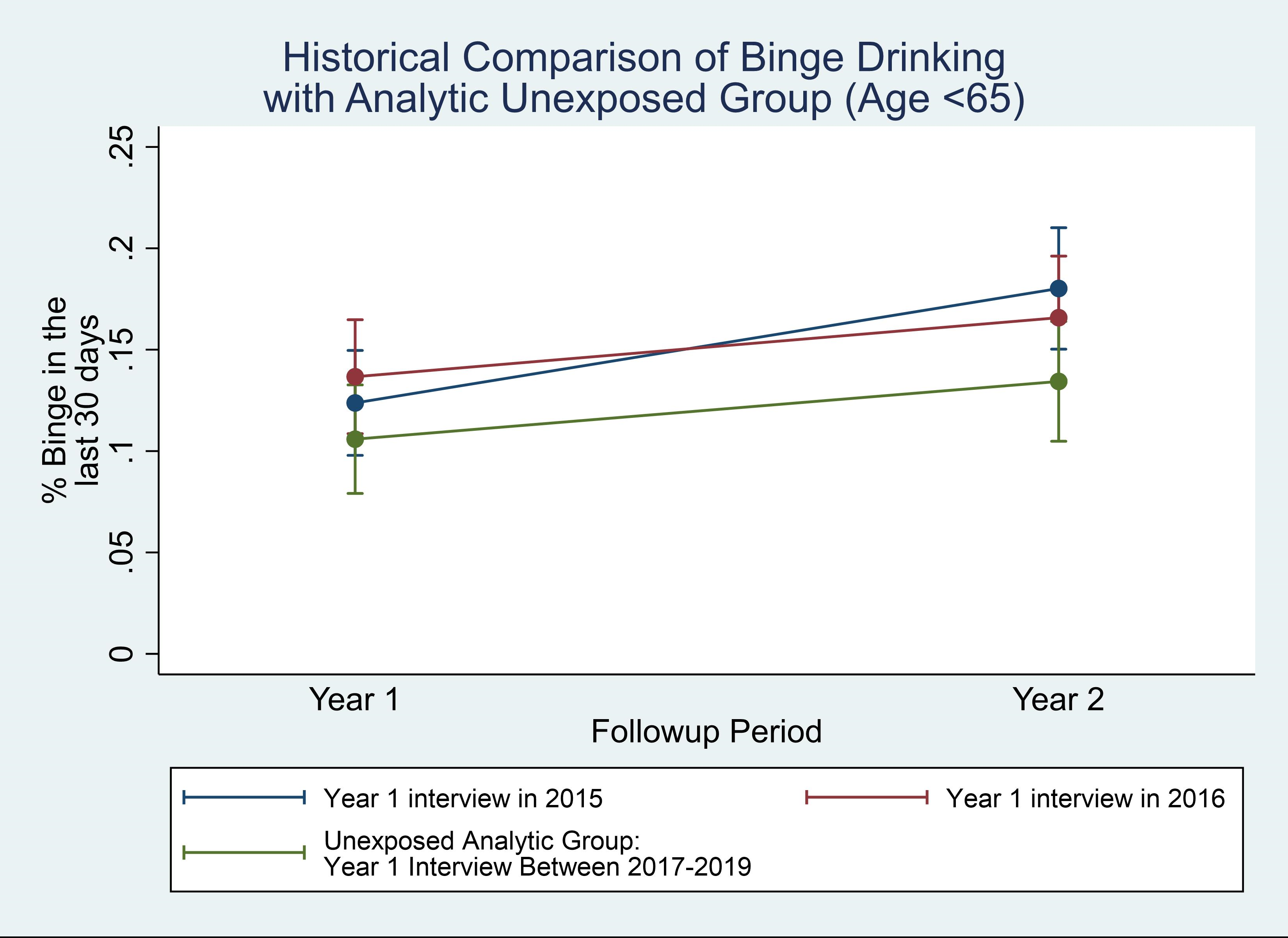

Supplement: S9 Fig — (TIF) [file pone.0266422.s010.tif]

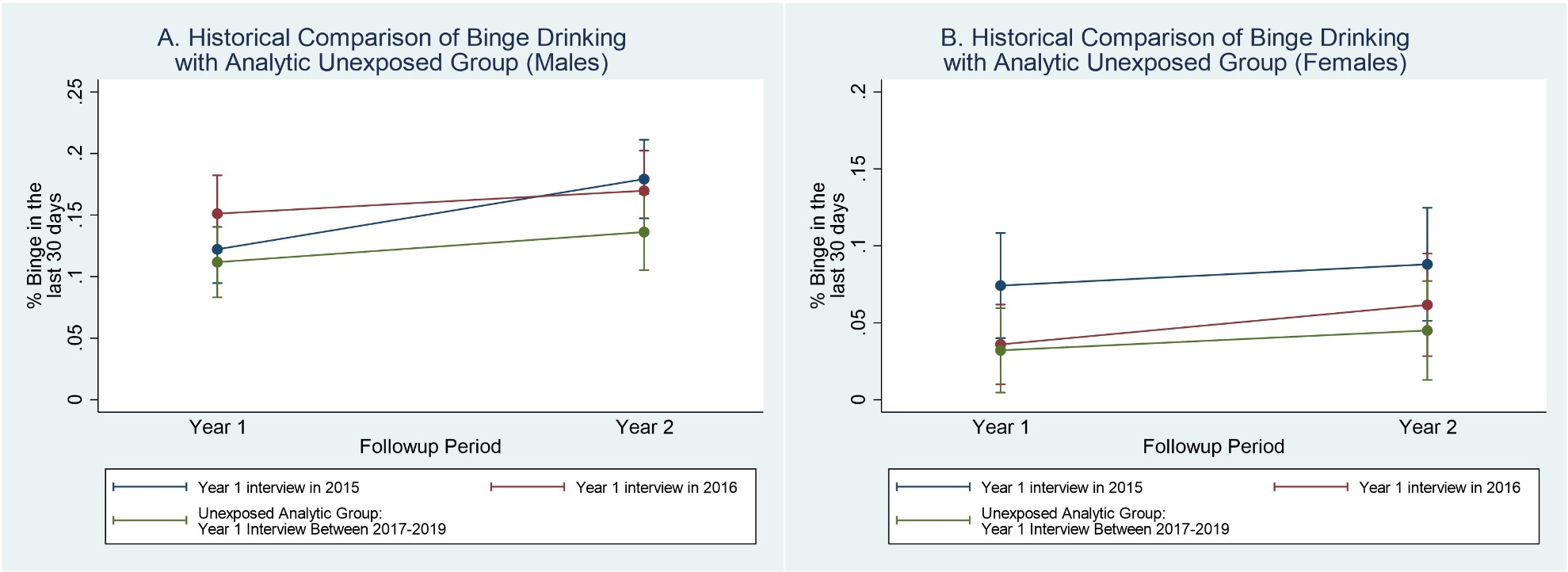

Supplement: S10 Fig — (TIF) [file pone.0266422.s011.tif]

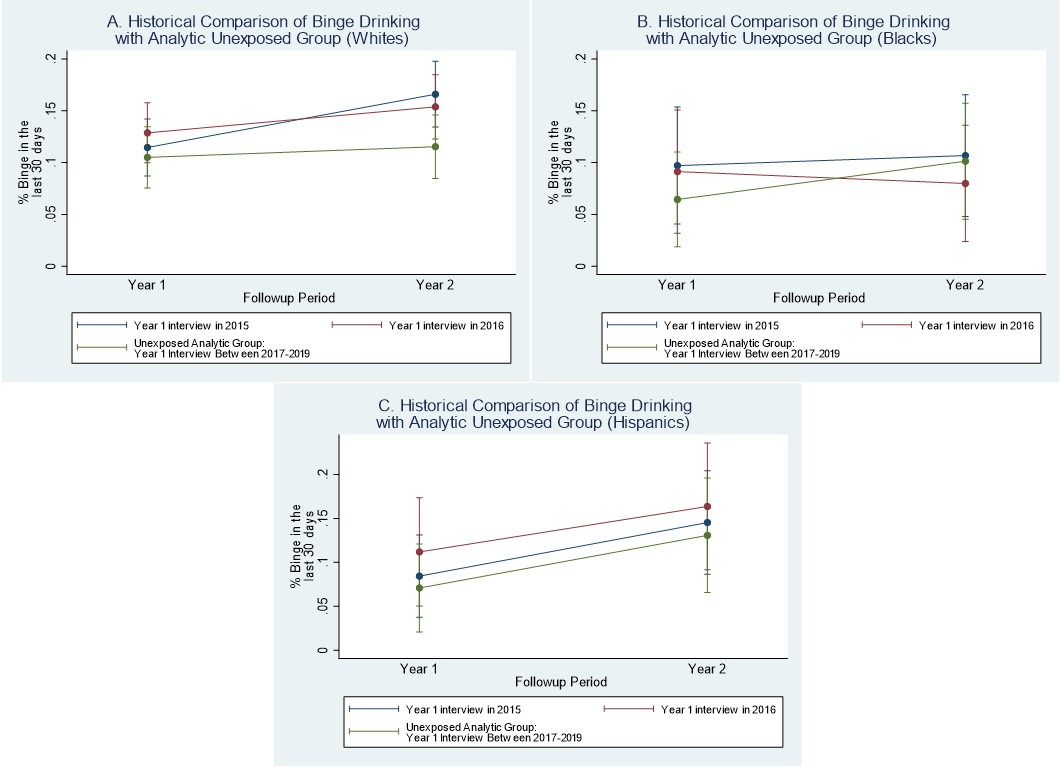

Supplement: S11 Fig — (TIF) [file pone.0266422.s012.tif]

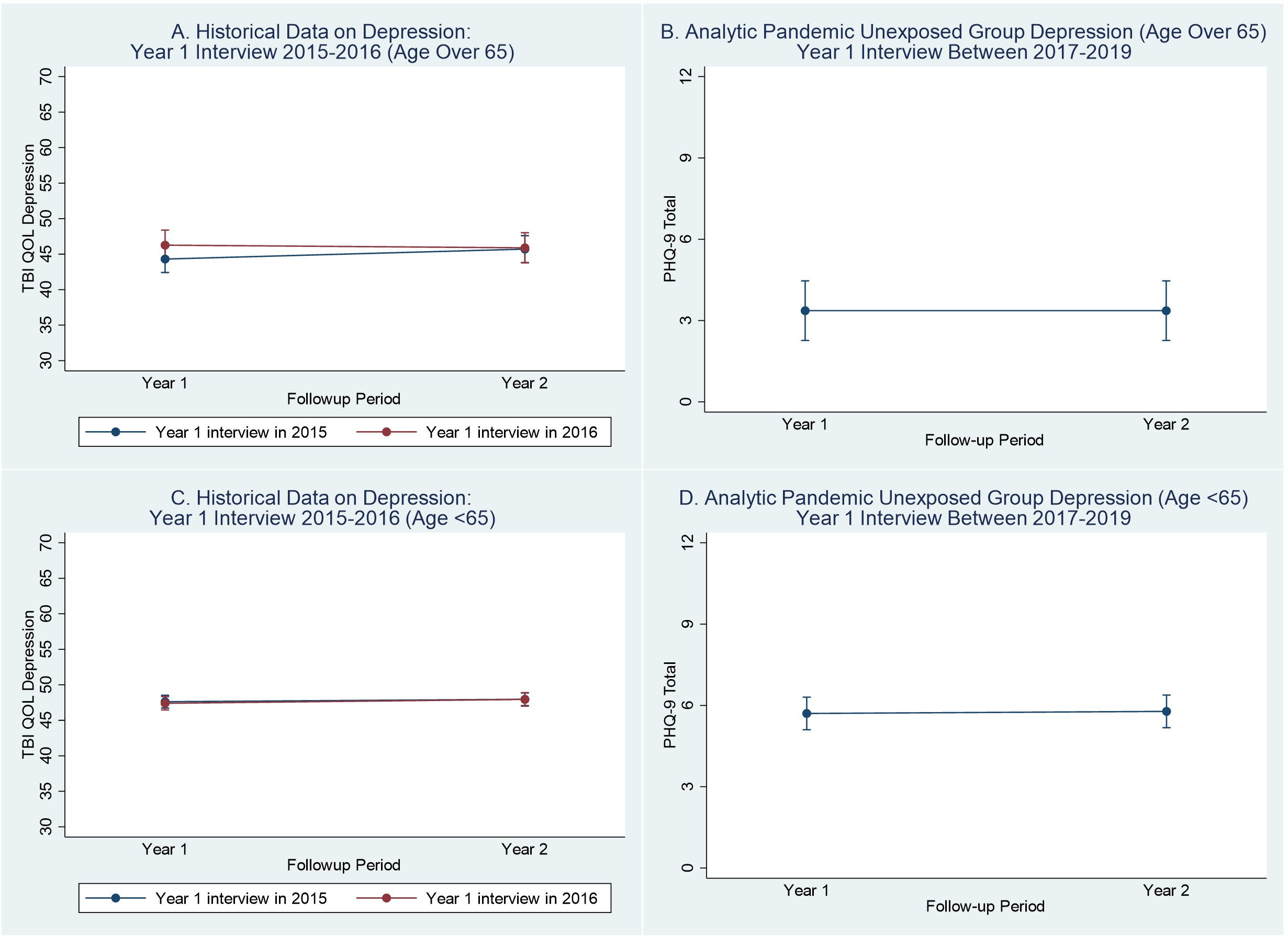

Supplement: S12 Fig — (TIF) [file pone.0266422.s013.tif]

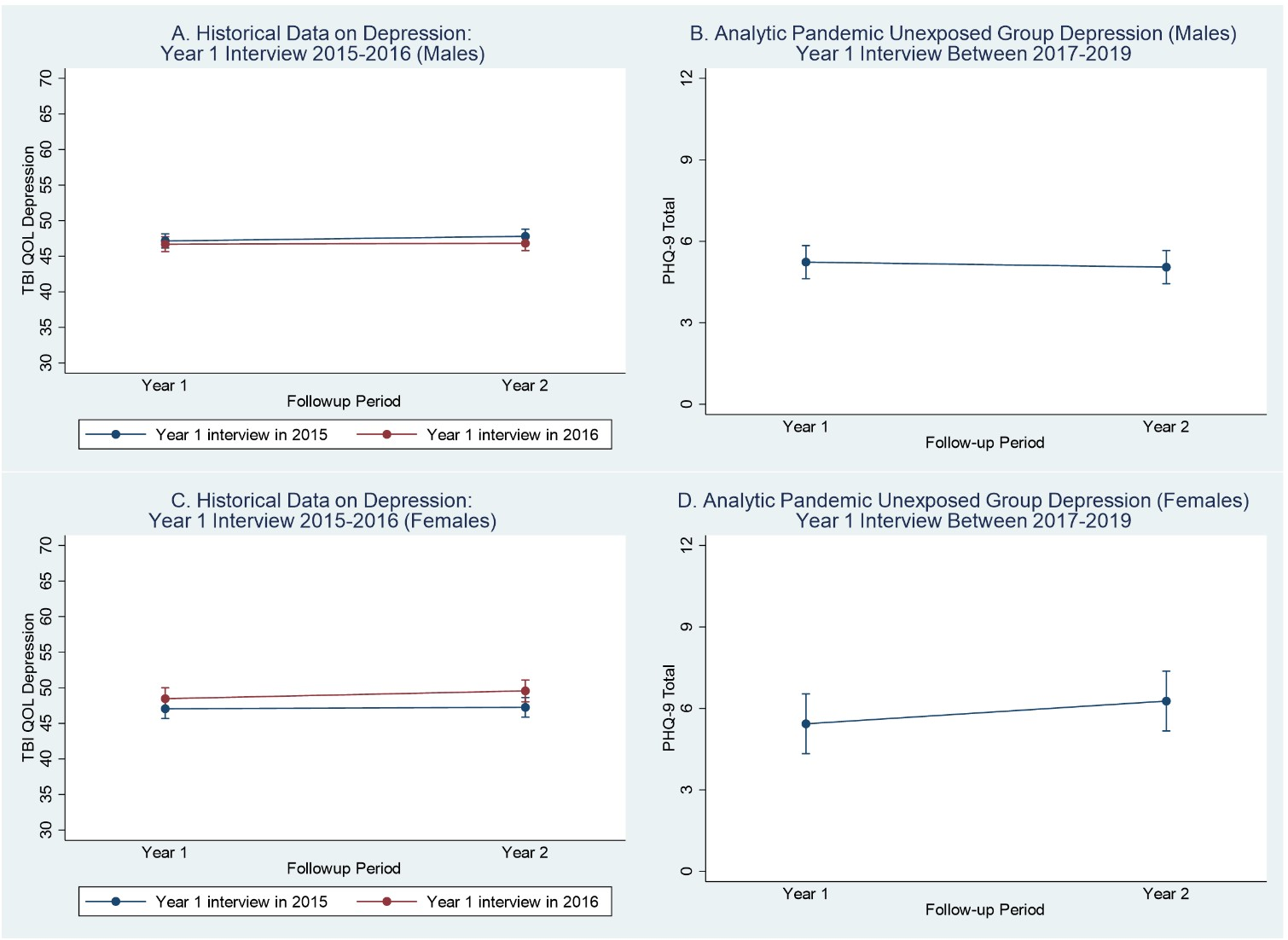

Supplement: S13 Fig — (TIF) [file pone.0266422.s014.tif]

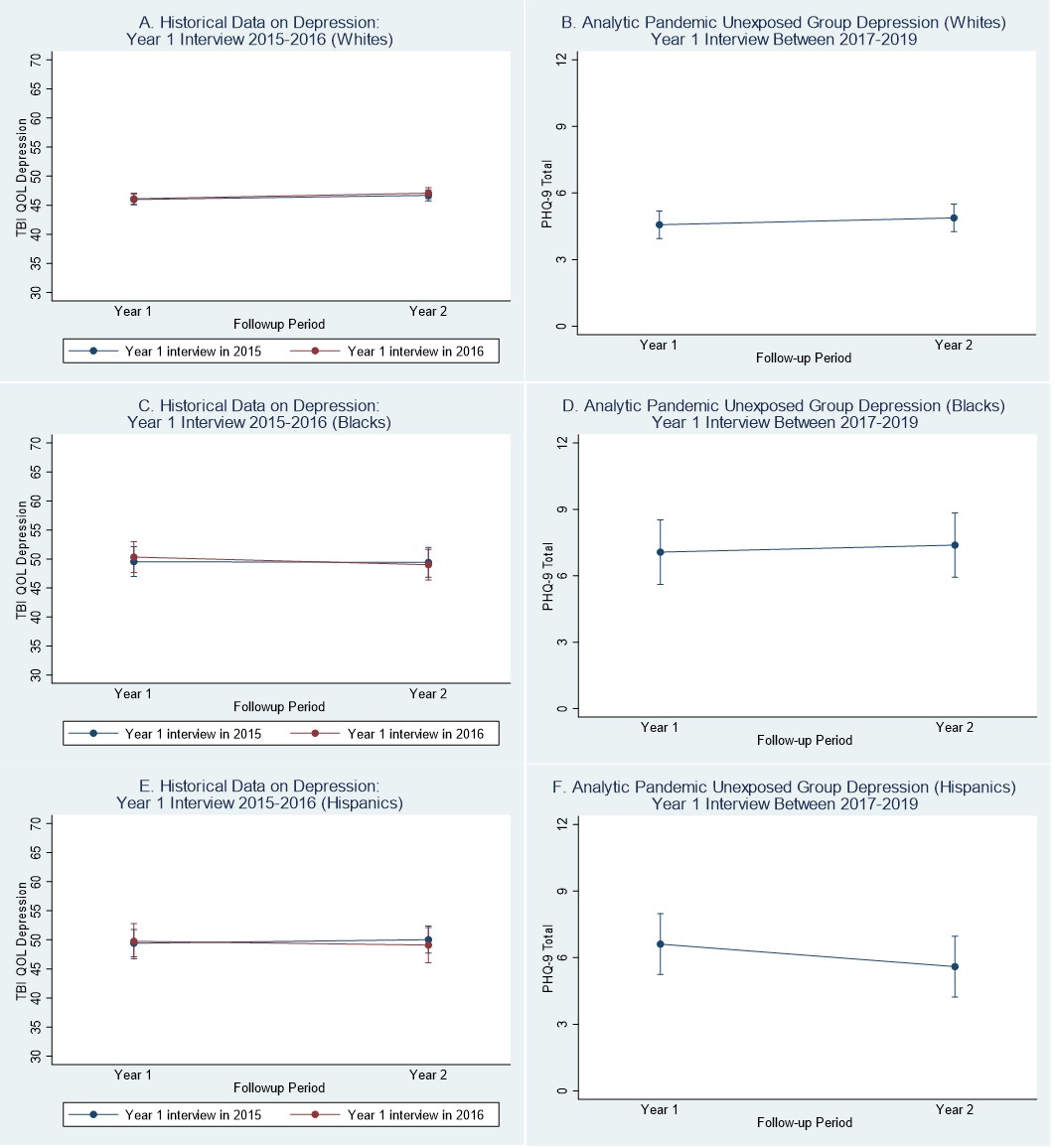

Supplement: S14 Fig — (TIF) [file pone.0266422.s015.tif]

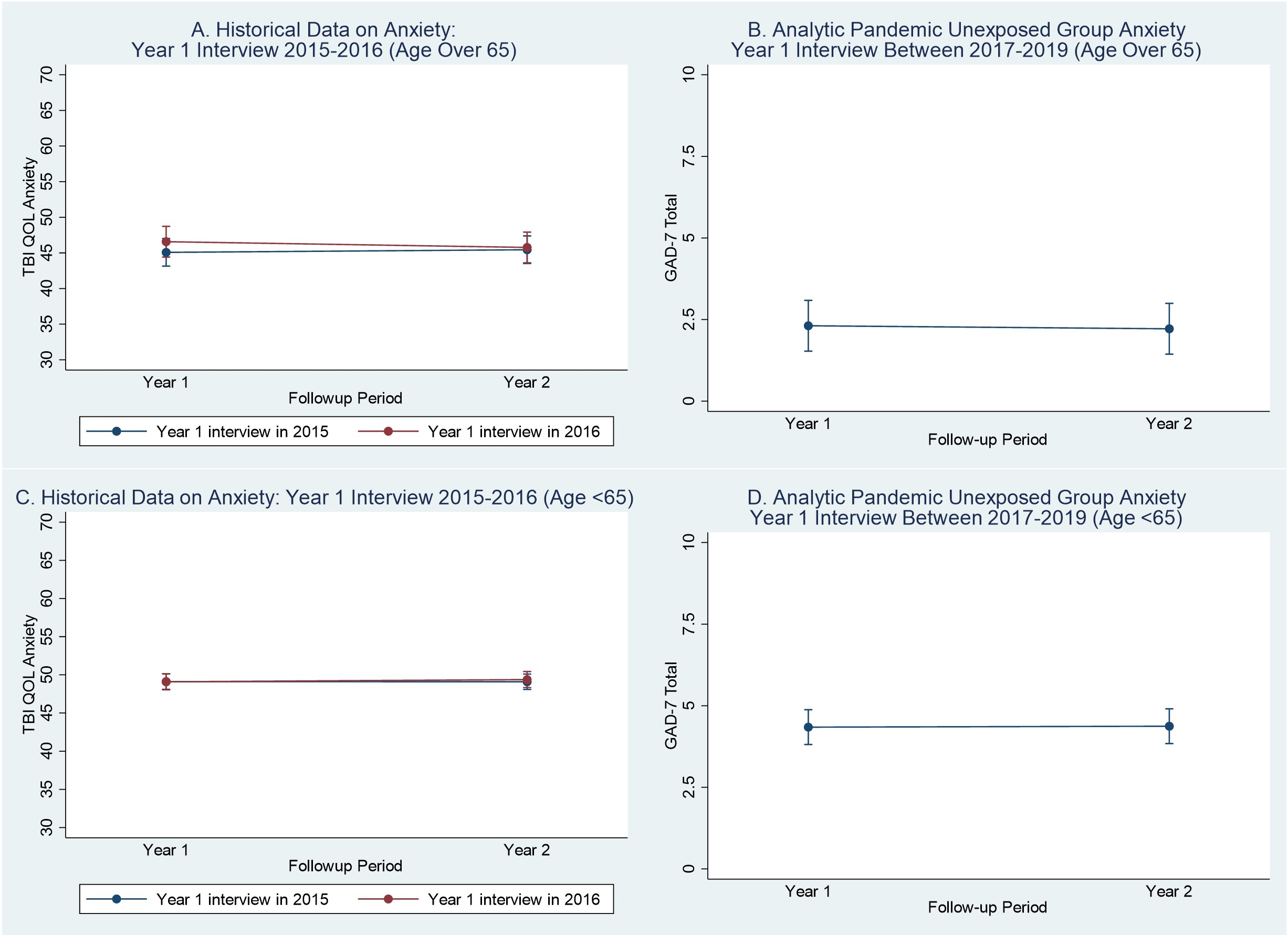

Supplement: S15 Fig — (TIF) [file pone.0266422.s016.tif]

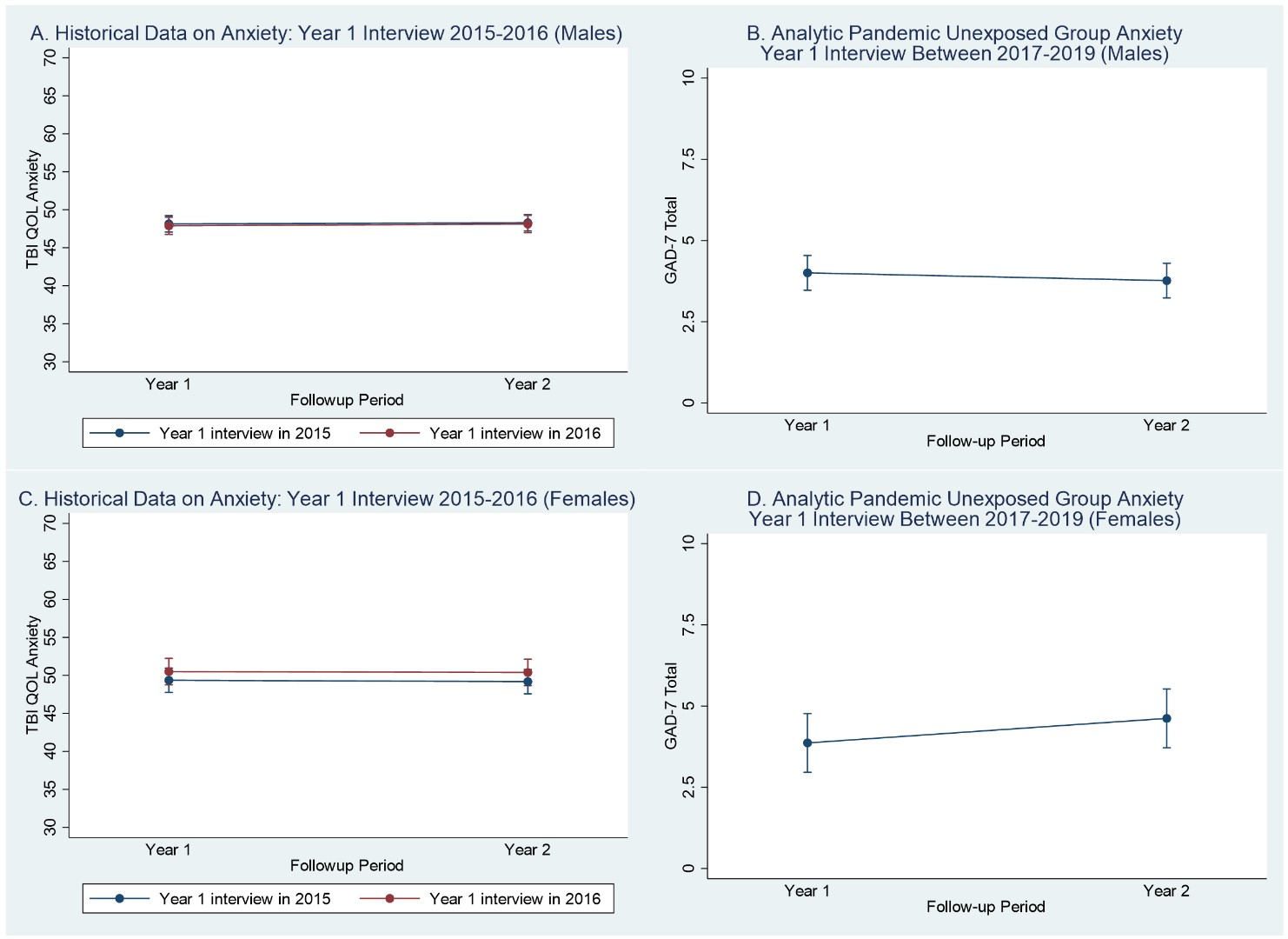

Supplement: S16 Fig — (TIF) [file pone.0266422.s017.tif]

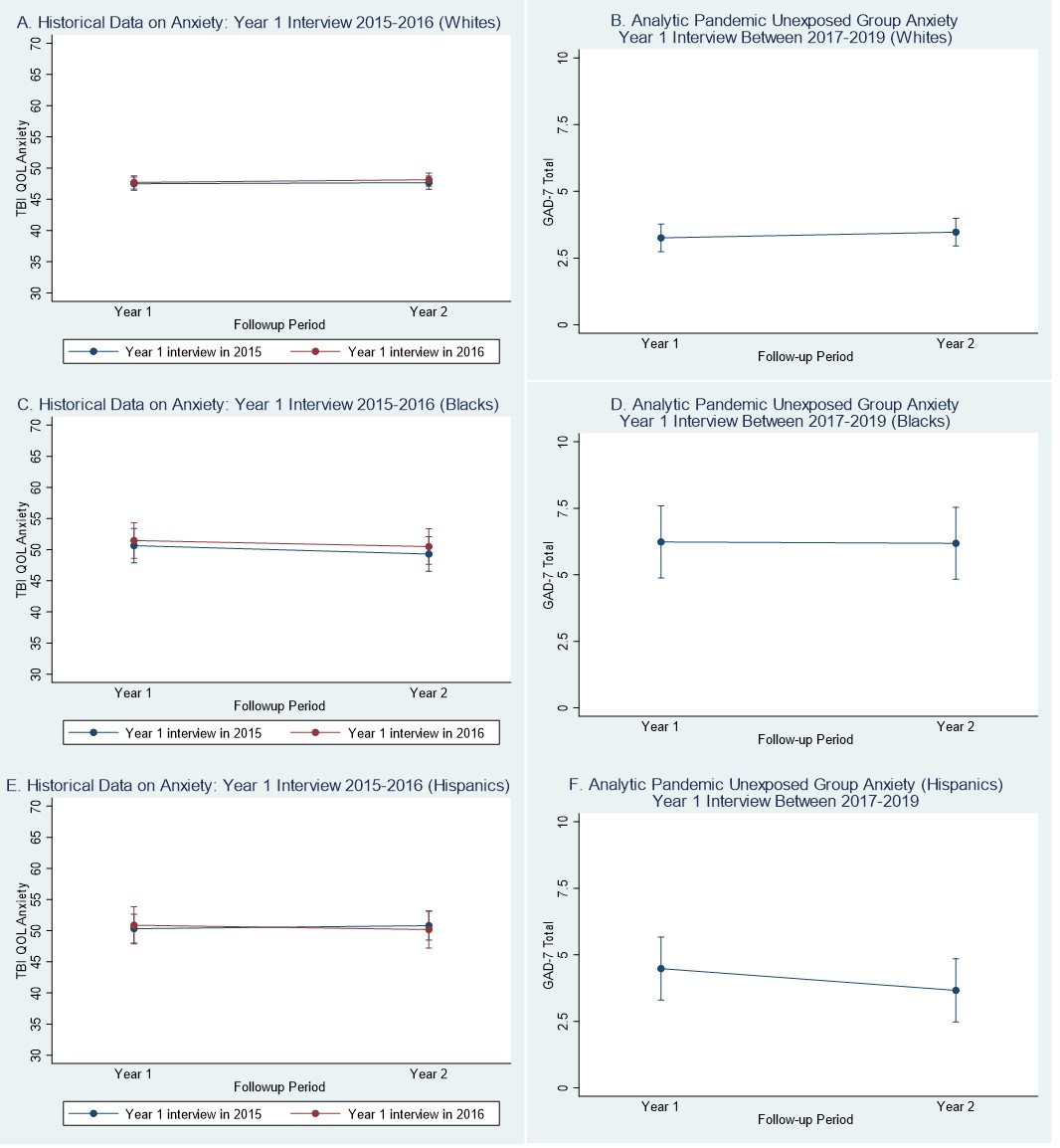

Supplement: S17 Fig — (TIF) [file pone.0266422.s018.tif]
