# Supplementary material for: Changes in alcohol use and mood during the COVID-19 pandemic among individuals with traumatic brain injury: A difference-in-difference study
Source: PLoS One. 2022 Apr 7;17(4):e0266422. doi: 10.1371/journal.pone.0266422 (PMC8989351; doi:10.1371/journal.pone.0266422)
Supplement: S1 Table — (DOCX) [file pone.0266422.s019.docx]

S1 Table: Investigation of Potential Selection Bias for Completed Year 2 Follow-up after Completed Year 1 Follow-up

|  | Analytic Sample^┼^ (n=1059) | Completed Year 1 interview, but did not complete Year 2 interview in window for any reason^§^ (n=235) | p-value |  |
| --- | --- | --- | --- | --- |
| **Demographic characteristics** | | | | |
| Age at injury, Mean (SD) | 46.2 (20.1) | 50.5 (21.8) | 0.009* |  |
| Sex, Men (%) | 784 (74.4%) | 165 (70.2%) | 0.190 |  |
| Race, n (%)  White  Black  Hispanic  Other | 682 (64.5%)  180 (17.0%)  139 (13.1%)  57 (5.4%) | 158 (67.5%)  37 (15.8%)  24 (10.3%)  15 (6.4%) | 0.560 |  |
| Education, n (%)  Less than HS  HS+ | 229 (21.8%)  822 (78.2%) | 63 (27.6%)  165 (72.4%) | 0.057 |  |
| Primary rehab payor source, n (%)  Private insurance  Medicare or Medicaid  Other | 434 (41.3%) 373 (35.5%)  244 (23.2%) | 86 (36.6%)  106 (45.1%)  43 (18.3%) | 0.019* |  |
| **Injury characteristics** | | | | |
| Mechanism of injury, n (%)  Motor vehicle  Fall  Any violence  Other | 391 (37.2%)  385 (36.6%)  74 (7.0%)  201 (19.1%) | 81 (34.5%)  111 (47.2%)  19 (8.1%)  24 (10.2%) | 0.002* |  |
| GCS score, Mean (SD) | 10.6 (4.5) | 11.1 (4.6) | 0.046* |  |
| TFC (days), Mean (SD) | 8.8 (23.8) | 6.2 (12.7) | 0.035* |  |
| Duration of PTA (days), Mean (SD) | 25.3 (32.1) | 22.9 (28.0) | 0.117 |  |
| Pre-index lifetime history of TBI, n (%) | 255 (24.3%) | 29 (13.4%) | <0.001* |  |
| **Clinical characteristics** | | | | |
| Acute hospital length of stay, Mean (SD) | 20.0 (17.2) | 17.1 (15.2) | <0.001* |  |
| Inpatient rehabilitation length of stay, Mean (SD) | 24.8 (26.1) | 22.3 (17.9) | 0.584 |  |
| Craniotomy or craniectomy, n (%) | 269 (25.6) | 64 (27.4) | 0.569 |  |
| FIM Motor at Rehabilitation discharge, Mean (SD) | 65.3 (17.8) | 63.9 (18.1) | 0.188 |  |
| FIM Cognitive at Rehabilitation Discharge, Mean (SD) | 23.7 (6.6) | 22.5 (6.8) | 0.013* |  |
| Residence after inpatient rehabilitation discharge, n (%)  Private residence  Nursing home/adult home  Other | 829 (78.8)  23 (2.2)  200 (19.0) | 173 (73.6)  1 (0.4)  61 (26.0) | 0.015* |  |
| **Year 1 characteristics** |  |  |  |  |
| PHQ-9, Mean (SD) | 5.5 (5.9) | 4.8 (5.0) | 0.464 |  |
| GAD-7, Mean (SD) | 4.0 (5.2) | 3.7 (5.0) | 0.713 |  |
| Any Alcohol Use in the last month, n (%) | 384 (37.1%) | 80 (36.7%) | 0.911 |  |
| Average number of drinks, Mean (SD) | 0.9 (1.7) | 0.9 (1.8) | 0.973 |  |
| Binge drinking in the last month, n (%) | 103 (10.2%) | 21 (9.7%) | 0.847 |  |

^┼^: Completed Year 1 and Year 2 follow-up interview within TBIMS SOP follow-up window; had year 1 interview after October 1^st^, 2017, and did not have Year 1 or Year 2 interview between January 1^st^, 2020-March 31^st^, 2020

^§^: Reasons for non-completion include: lost (n=92), refused (n=11), incarcerated (n=8), withdrew (n=34), death (n=54), or Year 2 interview out of the TBIMS window per SOP: outside +/- 3 months after 2 year anniversary of injury date (n=33)

*statistically significant at α=0.05
