# Supplementary material for: Changes in alcohol use and mood during the COVID-19 pandemic among individuals with traumatic brain injury: A difference-in-difference study
Source: PLoS One. 2022 Apr 7;17(4):e0266422. doi: 10.1371/journal.pone.0266422 (PMC8989351; doi:10.1371/journal.pone.0266422)
Supplement: S2 Table — (DOCX) [file pone.0266422.s020.docx]

S2 Table: Subgroup Difference-in-Difference Analyses of Average Number of Drinks by Pandemic Exposure Status

| Subgroup | COVID-19 pandemic exposure | Follow-up period | Mean^¥^ (SD) | DiD Parameter Estimate^┼^ (95% CI) | P-value |
| --- | --- | --- | --- | --- | --- |
| Age ≥ 65 | No (n=128) | Year 1 | 0.48 (0.78) | 0.19 (-0.33, 0.72) | 0.474 |
|  |  | Year 2 | 0.48 (0.75) |  |  |
|  | Yes (n=77) | Year 1 | 0.45 (0.85) |  |  |
|  |  | Year 2 | 0.56 (1.14) |  |  |
| Age < 65 | No (n=510) | Year 1 | 1.02 (2.00) | 0.40 (0.17, 0.63) | 0.001* |
|  |  | Year 2 | 1.13 (1.86) |  |  |
|  | Yes (n=258) | Year 1 | 0.88 (1.54) |  |  |
|  |  | Year 2 | 1.43 (2.28) |  |  |
| Males | No (n=476) | Year 1 | 1.00 (2.03) | 0.39 (0.16, 0.62) | 0.001* |
|  |  | Year 2 | 1.10 (1.90) |  |  |
|  | Yes (n=246) | Year 1 | 0.88 (1.56) |  |  |
|  |  | Year 2 | 1.42 (2.28) |  |  |
| Females | No (n=157) | Year 1 | 0.68 (1.02) | 0.20 (-0.27, 0.67) | 0.401 |
|  |  | Year 2 | 0.71 (0.95) |  |  |
|  | Yes (n=89) | Year 1 | 0.51 (0.89) |  |  |
|  |  | Year 2 | 0.71 (1.40) |  |  |
| White | No (n=410) | Year 1 | 1.01 (2.00) | 0.21 (-0.03, 0.45) | 0.089 |
|  |  | Year 2 | 1.06 (1.71) |  |  |
|  | Yes (n=222) | Year 1 | 0.86 (1.52) |  |  |
|  |  | Year 2 | 1.14 (1.88) |  |  |
| Black | No (n=113) | Year 1 | 0.73 (1.51) | 0.60 (0.01, 1.19) | 0.046* |
|  |  | Year 2 | 0.86 (1.86) |  |  |
|  | Yes (n=54) | Year 1 | 0.56 (1.14) |  |  |
|  |  | Year 2 | 1.17 (1.67) |  |  |
| Hispanic ethnicity | No (n=105) | Year 1 | 0.82 (1.48) | 0.48 (0.01, 0.96) | 0.045* |
|  |  | Year 2 | 1.01 (1.64) |  |  |
|  | Yes (n=49) | Year 1 | 0.71 (1.34) |  |  |
|  |  | Year 2 | 1.47 (3.22) |  |  |

^¥^: Descriptive measure, not model-based or adjusted for covariates

^┼^Estimate represents *pandemic exposure*followup period interaction* parameter estimate from GEE Model adjusted for age at injury, sex, race, and time to follow commands in days (interpreted as DiD in PHQ-9/GAD-7 between pandemic exposed vs. unexposed from year 1 to year 2)

*statistically significant at α=0.05
