# Supplementary material for: Changes in alcohol use and mood during the COVID-19 pandemic among individuals with traumatic brain injury: A difference-in-difference study
Source: PLoS One. 2022 Apr 7;17(4):e0266422. doi: 10.1371/journal.pone.0266422 (PMC8989351; doi:10.1371/journal.pone.0266422)
Supplement: S3 Table — (DOCX) [file pone.0266422.s021.docx]

S3 Table: Subgroup Difference-in-Difference Analyses of Any Alcohol Use in the last month by Pandemic Exposure Status

| Subgroup | COVID-19 pandemic exposure | Follow-up period | Ncases^¥^ (%) | DiD Parameter Estimate^┼^ (95% CI) | P-value |
| --- | --- | --- | --- | --- | --- |
| Age ≥ 65 | No (n=127) | Year 1 | 46 (36.2%) | 0.03 (-0.55, 0.61) | 0.912 |
|  |  | Year 2 | 47 (37.0%) |  |  |
|  | Yes (n=76) | Year 1 | 21 (27.6%) |  |  |
|  |  | Year 2 | 22 (29.0%) |  |  |
| Age < 65 | No (n=518) | Year 1 | 198 (38.2%) | 0.23 (-0.08, 0.55) | 0.142 |
|  |  | Year 2 | 229 (44.2%) |  |  |
|  | Yes (n=261) | Year 1 | 95 (36.4%) |  |  |
|  |  | Year 2 | 125 (47.9%) |  |  |
| Males | No (n=486) | Year 1 | 181 (37.2%) | 0.30 (-0.01, 0.61) | 0.058 |
|  |  | Year 2 | 204 (42.0%) |  |  |
|  | Yes (n=247) | Year 1 | 87 (35.2%) |  |  |
|  |  | Year 2 | 116 (47.0%) |  |  |
| Females | No (n=159) | Year 1 | 63 (39.6%) | -0.14 (-0.72, 0.43) | 0.621 |
|  |  | Year 2 | 72 (45.3%) |  |  |
|  | Yes (n=90) | Year 1 | 29 (32.2%) |  |  |
|  |  | Year 2 | 31 (34.4%) |  |  |
| White | No (n=415) | Year 1 | 172 (41.5%) | 0.04 (-0.28, 0.37) | 0.796 |
|  |  | Year 2 | 196 (47.2%) |  |  |
|  | Yes (n=224) | Year 1 | 84 (37.5%) |  |  |
|  |  | Year 2 | 99 (44.2%) |  |  |
| Black | No (n=114) | Year 1 | 37 (32.5%) | 0.55 (-0.17, 1.26) | 0.136 |
|  |  | Year 2 | 44 (38.6%) |  |  |
|  | Yes (n=54) | Year 1 | 14 (25.9%) |  |  |
|  |  | Year 2 | 23 (42.6%) |  |  |
| Hispanic ethnicity | No (n=105) | Year 1 | 37 (35.2%) | 0.22 (-0.46, 0.90) | 0.528 |
|  |  | Year 2 | 38 (36.2%) |  |  |
|  | Yes (n=51) | Year 1 | 17 (33.3%) |  |  |
|  |  | Year 2 | 20 (39.2%) |  |  |

^¥^: Descriptive measure, not model-based or adjusted for covariates

^┼^Estimate represents *pandemic exposure*followup period interaction* parameter estimate from GEE Model adjusted for age at injury, sex (unless sex subgroup analysis), race (unless race subgroup analysis), and time to follow commands in days (interpreted as DiD in PHQ-9/GAD-7 between pandemic exposed vs. unexposed from year 1 to year 2)
