# Supplementary material for: Changes in alcohol use and mood during the COVID-19 pandemic among individuals with traumatic brain injury: A difference-in-difference study
Source: PLoS One. 2022 Apr 7;17(4):e0266422. doi: 10.1371/journal.pone.0266422 (PMC8989351; doi:10.1371/journal.pone.0266422)
Supplement: S4 Table — (DOCX) [file pone.0266422.s022.docx]

S4 Table: Subgroup Difference-in-Difference Analyses of Binge Drinking in the last month by Pandemic Exposure Status

| Subgroup | COVID-19 pandemic exposure | Follow-up period | Ncases^¥^ (%) | DiD Parameter Estimate^┼^ (95% CI) | P-value |
| --- | --- | --- | --- | --- | --- |
| Age ≥ 65 | No (n=123) | Year 1 | 3 (2.4%) | n/a^§^ | n/a^§^ |
|  |  | Year 2 | 3 (2.4%) |  |  |
|  | Yes (n=76) | Year 1 | 3 (4.0%) |  |  |
|  |  | Year 2 | 0 (0%) |  |  |
| Age < 65 | No (n=506) | Year 1 | 60 (11.9%) | 0.14 (-0.35, 0.64) | 0.570 |
|  |  | Year 2 | 74 (14.6%) |  |  |
|  | Yes (n=255) | Year 1 | 28 (11.0%) |  |  |
|  |  | Year 2 | 39 (15.3%) |  |  |
| Males | No (n=472) | Year 1 | 50 (10.6%) | 0.15 (-0.40, 0.71) | 0.588 |
|  |  | Year 2 | 61 (12.9%) |  |  |
|  | Yes (n=242) | Year 1 | 23 (9.5%) |  |  |
|  |  | Year 2 | 32 (13.2%) |  |  |
| Females | No (n=157) | Year 1 | 13 (8.3%) | -0.41 (-1.66, 0.83) | 0.514 |
|  |  | Year 2 | 16 (10.2%) |  |  |
|  | Yes (n=89) | Year 1 | 8 (9.0%) |  |  |
|  |  | Year 2 | 7 (7.9%) |  |  |
| White | No (n=405) | Year 1 | 48 (11.9%) | 0.08 (-0.50, 0.65) | 0.791 |
|  |  | Year 2 | 53 (13.1%) |  |  |
|  | Yes (n=221) | Year 1 | 22 (10.0%) |  |  |
|  |  | Year 2 | 26 (11.8%) |  |  |
| Black | No (n=111) | Year 1 | 7 (6.3%) | 0.90 (-0.73, 2.52) | 0.280 |
|  |  | Year 2 | 11 (9.9%) |  |  |
|  | Yes (n=52) | Year 1 | 2 (3.9%) |  |  |
|  |  | Year 2 | 7 (13.5%) |  |  |
| Hispanic ethnicity | No (n=103) | Year 1 | 9 (8.7%) | -0.96 (-2.25, 0.34) | 0.148 |
|  |  | Year 2 | 14 (13.6%) |  |  |
|  | Yes (n=49) | Year 1 | 6 (12.2%) |  |  |
|  |  | Year 2 | 4 (8.2%) |  |  |

^¥^: Descriptive measure, not model-based or adjusted for covariates

^┼^Estimate represents *pandemic exposure*followup period interaction* parameter estimate from GEE Model adjusted for age at injury, sex, race, and time to follow commands in days (interpreted as DiD in PHQ-9/GAD-7 between pandemic exposed vs. unexposed from year 1 to year 2

^§^GEE Model did not converge, too few cases
