# Supplementary material for: Changes in alcohol use and mood during the COVID-19 pandemic among individuals with traumatic brain injury: A difference-in-difference study
Source: PLoS One. 2022 Apr 7;17(4):e0266422. doi: 10.1371/journal.pone.0266422 (PMC8989351; doi:10.1371/journal.pone.0266422)
Supplement: S7 Table — (DOCX) [file pone.0266422.s025.docx]

S7 Table: Sensitivity Analysis of Alcohol Use Outcomes Excluding Pre-injury Abstainers

| Outcome |  | | | | |
| --- | --- | --- | --- | --- | --- |
| Any alcohol use in the last month | COVID-19 pandemic exposure | Follow-up period | Ncases^¥^ (%) | DiD Parameter Estimate^§^ (95% CI) | P-value |
|  | No (n=652) | Year 1 | 192 (49.6%) | 0.24 (-0.11, 0.60) | 0.177 |
|  |  | Year 2 | 208 (53.8%) |  |  |
|  | Yes (n=343) | Year 1 | 96 (47.3%) |  |  |
|  |  | Year 2 | 116 (57.1%) |  |  |
| Average number of drinks per occasion | COVID-19 pandemic exposure | Follow-up period | Mean^¥^ (SE) | DiD Parameter Estimate^€^ (95% CI) | P-value |
|  | No (n=638) | Year 1 | 1.27 (2.14) | 0.39 (0.14, 0.65) | 0.002* |
|  |  | Year 2 | 1.31 (1.86) |  |  |
|  | Yes (n=335) | Year 1 | 1.08 (1.59) |  |  |
|  |  | Year 2 | 1.62 (2.15) |  |  |
| Any binge drinking in the last month | COVID-19 pandemic exposure | Follow-up period | Ncases^¥^ (%) | DiD Parameter Estimate^§^ (95% CI) | P-value |
|  | No (n=631) | Year 1 | 49 (13.3%) | -0.07 (-0.62, 0.49) | 0.816 |
|  |  | Year 2 | 63 (17.1%) |  |  |
|  | Yes (n=337) | Year 1 | 28 (14.0%) |  |  |
|  |  | Year 2 | 34 (17.0%) |  |  |

^¥^Descriptive measure, not model-based or adjusted for covariates

^§^Estimate represents *pandemic exposure*followup period interaction* parameter estimate from GEE Model with binomial distribution and logit link. The GEE model adjusted for age at injury, sex, race, and time to follow commands in days (interpreted as DiD in any alcohol use/any binge drinking between pandemic exposed vs. unexposed from year 1 to year 2)

^€^Estimate represents *pandemic exposure*followup period interaction* parameter estimate from GEE Model with negative binomial distribution and log link. The GEE model adjusted for age at injury, sex, race, and time to follow commands in days (interpreted as DiD in average number of drinks consumed per occasion between pandemic exposed vs. unexposed from year 1 to year 2)
