# Supplementary material for: Changes in alcohol use and mood during the COVID-19 pandemic among individuals with traumatic brain injury: A difference-in-difference study
Source: PLoS One. 2022 Apr 7;17(4):e0266422. doi: 10.1371/journal.pone.0266422 (PMC8989351; doi:10.1371/journal.pone.0266422)
Supplement: Attachment — Submitted filename: Response to reviews FINAL.docx [file pone.0266422.s026.docx]

**Response to reviews:**

**Reviewer #1: This is an interesting analysis and obviously timely. I have only one significant comment and it is a criticism but something that should be discussed. In the data presented on the Y1-Y2 differences in number of drinks the significant DID seems to be mediated by both a larger number of Y2 drinks and a smaller number of Y1 for the pandemic exposed individuals. Is this apparent difference statistically significant, what could have caused it and would controlling for it reduce the effect of the pandemic?**

**RESPONSE:** This reviewer raises an important question that our collaborative group discussed at length when devising our analytic strategy. We followed a rigorous methodology consistent with difference-in-difference analyses to test model assumptions as recommended by Wing et al. (2018). The main assumption with these models is the “parallel trend assumption” which presupposes that the trend over time observed in the unexposed group *would have been* observed in our exposed group had they not been exposed. We used historical data collected in the TBI Model Systems from 2015-2016 (i.e., preceding the timeframe of our analytic sample) to test our parallel trend assumption for the primary analyses and all subgroup analyses (see **Supplemental Figures 1-17**). The main parameter of interest in difference-in-difference analyses is the interaction term (β_3_) in the general equation below.

*E(Y) = β_0_ + β_1_(Pandemic Exposed) + β_2_(Time) + β_3_(Exposed*Time)* + ɛ

Crucially, while these longitudinal models *do* already account for the baseline main effects of exposure status (*β_1_*), it is not required that *β_1_* be non-significant for there to be an observed difference between exposure groups over time (e.g., β_3_). Therefore, any small difference in average number of drinks at baseline (0.91 unexposed vs. 0.78 exposed) do not change the conclusions regarding the impact of the pandemic observed herein. To make this point clearer, we have now clarified in our Methods section that the parallel trend assumption does not presuppose that exposure groups be balanced with respect the outcome at baseline (p. 8 lines 205-208). We have also provided the reference below that provides further detail that may be of interest.

**Reference:**

Wing C, Simon K, Bello-Gomez RA. Designing difference in difference studies: best practices for public health policy research. Annual review of public health. 2018;39.

**Reviewer #2: This manuscript uses a unique and large dataset in order to answer an important question - what is the impact of experiencing the COVID-19 pandemic on alcohol consumption and is this different for subgroups of people with known COVID-19 vulnerabilities? That cannot be overstated. However, there are a few concerns that I have with the manuscript that dampen enthusiasm.**

**- The alcohol consumption measures are limited to drinks per occasion and binge drinking. Validated measures of alcohol consumption or an index of alcohol use disorder are not included in this dataset. This should be stated as a limitation in the discussion.**

**RESPONSE:** We thank this reviewer for this comment to allow us the opportunity to clarify details on the strengths and limitations of our alcohol data collection in this study. In 2017, the TBIMS National Database changed their data collection protocol to be consistent with the National Institute on Alcohol Abuse and Alcoholism (NIAAA) guidelines for binge drinking, which are 5+ drinks on an occasion for men, and 4+ drinks on an occasion for women. This NIAAA sex-specific binge drinking definition was applied in our full sample, which we have described in our methods section (p. 6 lines 149-151). That said, this reviewer’s point is well-taken, and unfortunately we did not have information available in this study on alcohol use disorder, nor measures related to consequences of drinking. We have now added these points as limitations in our discussion (p. 14 lines 360-362).

**- The 3rd paragraph of the discussion around the self-medication hypothesis is entirely and highly speculative given the data available and recommend removing entirely. I am not sure that perpetuation of a self-medication hypothesis in the substance use disorder field is helpful.**

**RESPONSE:** As suggested by the reviewer, we have removed discussion of the self-medication hypothesis in the discussion (p. 12 lines 307-310).

**- The mental health measures used necessitate changing references to anxiety and depression to anxiety and depression symptoms as they are not diagnostic in nature.**

**RESPONSE:** We thank this reviewer for this comment. We agree and have gone through our entire manuscript and made changes to the language such that anxiety and depression are now referred to as anxiety symptoms and depressive symptoms, respectively.

**- The data figures (3 and 4) are not legible and need to be improved.**

**RESPONSE:** We thank the reviewer for this feedback. We have now split up Figures 3 and 4 into multiple figures, which substantially reduces the number of panels in each figure. We have also enhanced the quality of each figure to further aid in legibility.
