# Supplementary material for: Changes in alcohol use and mood during the COVID-19 pandemic among individuals with traumatic brain injury: A difference-in-difference study
Source: PLoS One. 2022 Apr 7;17(4):e0266422. doi: 10.1371/journal.pone.0266422 (PMC8989351; doi:10.1371/journal.pone.0266422)
Supplement: Peer review history

# Peer review: Decision Letter 0

**DOI:** 10.1371/journal.pone.0266422.r001

**Authors:** Mohammad Farris Iman Leong Bin Abdullah

3 Feb 2022

PONE-D-21-37809Changes in Alcohol Use and Mood during the COVID-19 Pandemic among Individuals with Traumatic Brain Injury: A Difference-in-Difference StudyPLOS ONE

Dear Dr. Kumar,

Thank you for submitting your manuscript to PLOS ONE. After careful consideration, we feel that it has merit but does not fully meet PLOS ONE’s publication criteria as it currently stands. Therefore, we invite you to submit a revised version of the manuscript that addresses the points raised during the review process.

Please submit your revised manuscript by Mar 20 2022 11:59PM. If you will need more time than this to complete your revisions, please reply to this message or contact the journal office at plosone@plos.org. When you're ready to submit your revision, log on to https://www.editorialmanager.com/pone/ and select the 'Submissions Needing Revision' folder to locate your manuscript file.

Please include the following items when submitting your revised manuscript:

- A rebuttal letter that responds to each point raised by the academic editor and reviewer(s). You should upload this letter as a separate file labeled 'Response to Reviewers'.
- A marked-up copy of your manuscript that highlights changes made to the original version. You should upload this as a separate file labeled 'Revised Manuscript with Track Changes'.
- An unmarked version of your revised paper without tracked changes. You should upload this as a separate file labeled 'Manuscript'.

If you would like to make changes to your financial disclosure, please include your updated statement in your cover letter. Guidelines for resubmitting your figure files are available below the reviewer comments at the end of this letter.

If applicable, we recommend that you deposit your laboratory protocols in protocols.io to enhance the reproducibility of your results. Protocols.io assigns your protocol its own identifier (DOI) so that it can be cited independently in the future. For instructions see: https://journals.plos.org/plosone/s/submission-guidelines#loc-laboratory-protocols. Additionally, PLOS ONE offers an option for publishing peer-reviewed Lab Protocol articles, which describe protocols hosted on protocols.io. Read more information on sharing protocols at https://plos.org/protocols?utm_medium=editorial-email&utm_source=authorletters&utm_campaign=protocols.

We look forward to receiving your revised manuscript.

Kind regards,

Mohammad Farris Iman Leong Bin Abdullah, Dr Psych

Academic Editor

PLOS ONE

Journal Requirements:

When submitting your revision, we need you to address these additional requirements.

1. Please ensure that your manuscript meets PLOS ONE's style requirements, including those for file naming. The PLOS ONE style templates can be found at 

https://journals.plos.org/plosone/s/file?id=wjVg/PLOSOne_formatting_sample_main_body.pdf and 

https://journals.plos.org/plosone/s/file?id=ba62/PLOSOne_formatting_sample_title_authors_affiliations.pdf

2. Thank you for stating in your Funding Statement: 

Dr. Dams-O’Connor, Dr. Kumar, and Dr. Yew’s effort were support in part by a grant from National Institute on Disability Independent Living and Rehabilitation Research (NIDILRR) to the Icahn School of Medicine at Mount Sinai (90DP0038 and 90DPTB0009). Dr. Kumar was also supported in part by a grant from the National Institute of Health’s Eunice Kennedy Shriver National Institute of Child Health and Human Development (1K99HD106060-01). Dr. Dams-O’Connor is also supported in part by a grant from the National Institute of Health’s National Institute on Neurological Disorders and Stroke (RF1NS115268). Dr. Dreer’s support was funded in part by a NIDILRR grant to the Department of Physical Medicine & Rehabilitation in the School of Medicine at the University of Alabama at Birmingham Rehabilitation, University of Alabama at Birmingham (NIDILRR: 90DPTB0015). Dr. Esterov’s support was funded in part by a NIDILRR grant to the Department of Physical Medicine and Rehabilitation at Mayo Clinic, (NIDILRR: 90DPTB0012-01-00). Dr. Chiaravalloti’s support was funded by a NIDILRR grant to Kessler Foundation (NIDILRR: 90DPTB0003). Dr. Corrigan’s effort was supported in part by a grant from NIDILRR to Ohio State University (90DPTB0001). Dr. Juengst’s effort was supported in part by a grant from NIDILRR to the University of Texas Southwestern Medical Center (90DPTB0013) and TIRR Memorial Hermann Hospital (90DPTB0016).

NIDILRR is a Center within the Administration for Community Living (ACL), Department of Health and Human Services (HHS). The contents of this publication do not necessarily represent the policy of NIH, NIDILRR, the Administration on Community Living, the U.S. Department of Health and Human Services, the U.S. Department of Veterans Affairs, and endorsement by the Federal Government should not be assumed.  

Please provide an amended statement that declares *all* the funding or sources of support (whether external or internal to your organization) received during this study, as detailed online in our guide for authors at http://journals.plos.org/plosone/s/submit-now.  Please also include the statement “There was no additional external funding received for this study.” in your updated Funding Statement. 

Please include your amended Funding Statement within your cover letter. We will change the online submission form on your behalf.

3. Thank you for stating the following financial disclosure: 

Dr. Dams-O’Connor, Dr. Kumar, and Dr. Yew’s effort were support in part by a grant from National Institute on Disability Independent Living and Rehabilitation Research (NIDILRR) to the Icahn School of Medicine at Mount Sinai (90DP0038 and 90DPTB0009). Dr. Kumar was also supported in part by a grant from the National Institute of Health’s Eunice Kennedy Shriver National Institute of Child Health and Human Development (1K99HD106060-01). Dr. Dams-O’Connor is also supported in part by a grant from the National Institute of Health’s National Institute on Neurological Disorders and Stroke (RF1NS115268). Dr. Dreer’s support was funded in part by a NIDILRR grant to the Department of Physical Medicine & Rehabilitation in the School of Medicine at the University of Alabama at Birmingham Rehabilitation, University of Alabama at Birmingham (NIDILRR: 90DPTB0015). Dr. Esterov’s support was funded in part by a NIDILRR grant to the Department of Physical Medicine and Rehabilitation at Mayo Clinic, (NIDILRR: 90DPTB0012-01-00). Dr. Chiaravalloti’s support was funded by a NIDILRR grant to Kessler Foundation (NIDILRR: 90DPTB0003). Dr. Corrigan’s effort was supported in part by a grant from NIDILRR to Ohio State University (90DPTB0001). Dr. Juengst’s effort was supported in part by a grant from NIDILRR to the University of Texas Southwestern Medical Center (90DPTB0013) and TIRR Memorial Hermann Hospital (90DPTB0016).

NIDILRR is a Center within the Administration for Community Living (ACL), Department of Health and Human Services (HHS). The contents of this publication do not necessarily represent the policy of NIH, NIDILRR, the Administration on Community Living, the U.S. Department of Health and Human Services, the U.S. Department of Veterans Affairs, and endorsement by the Federal Government should not be assumed.  

  

Please state what role the funders took in the study.  If the funders had no role, please state: "The funders had no role in study design, data collection and analysis, decision to publish, or preparation of the manuscript." 

If this statement is not correct you must amend it as needed. 

Please include this amended Role of Funder statement in your cover letter; we will change the online submission form on your behalf.

4. Thank you for stating the following in the Competing Interests section: 

Dr. Adams consults for TIAG, in support of the National Intrepid Center of Excellence (NICOoE) at Walter Reed National Medical Center.

Please confirm that this does not alter your adherence to all PLOS ONE policies on sharing data and materials, by including the following statement: "This does not alter our adherence to  PLOS ONE policies on sharing data and materials.” (as detailed online in our guide for authors http://journals.plos.org/plosone/s/competing-interests).  If there are restrictions on sharing of data and/or materials, please state these. Please note that we cannot proceed with consideration of your article until this information has been declared. 

Please include your updated Competing Interests statement in your cover letter; we will change the online submission form on your behalf.

5. In your Data Availability statement, you have not specified where the minimal data set underlying the results described in your manuscript can be found. PLOS defines a study's minimal data set as the underlying data used to reach the conclusions drawn in the manuscript and any additional data required to replicate the reported study findings in their entirety. All PLOS journals require that the minimal data set be made fully available. For more information about our data policy, please see http://journals.plos.org/plosone/s/data-availability.

Upon re-submitting your revised manuscript, please upload your study’s minimal underlying data set as either Supporting Information files or to a stable, public repository and include the relevant URLs, DOIs, or accession numbers within your revised cover letter. For a list of acceptable repositories, please see http://journals.plos.org/plosone/s/data-availability#loc-recommended-repositories. Any potentially identifying patient information must be fully anonymized.

Important: If there are ethical or legal restrictions to sharing your data publicly, please explain these restrictions in detail. Please see our guidelines for more information on what we consider unacceptable restrictions to publicly sharing data: http://journals.plos.org/plosone/s/data-availability#loc-unacceptable-data-access-restrictions. Note that it is not acceptable for the authors to be the sole named individuals responsible for ensuring data access.

We will update your Data Availability statement to reflect the information you provide in your cover letter.

6. Please include captions for your Supporting Information files at the end of your manuscript, and update any in-text citations to match accordingly. Please see our Supporting Information guidelines for more information: http://journals.plos.org/plosone/s/supporting-information. 

7. Please review your reference list to ensure that it is complete and correct. If you have cited papers that have been retracted, please include the rationale for doing so in the manuscript text, or remove these references and replace them with relevant current references. Any changes to the reference list should be mentioned in the rebuttal letter that accompanies your revised manuscript. If you need to cite a retracted article, indicate the article’s retracted status in the References list and also include a citation and full reference for the retraction notice.

Reviewers' comments:

Reviewer's Responses to Questions

**Comments to the Author**

1. Is the manuscript technically sound, and do the data support the conclusions?

The manuscript must describe a technically sound piece of scientific research with data that supports the conclusions. Experiments must have been conducted rigorously, with appropriate controls, replication, and sample sizes. The conclusions must be drawn appropriately based on the data presented. 

Reviewer #1: Yes

Reviewer #2: Yes

**********

2. Has the statistical analysis been performed appropriately and rigorously? 

Reviewer #1: Yes

Reviewer #2: Yes

**********

3. Have the authors made all data underlying the findings in their manuscript fully available?

The PLOS Data policy requires authors to make all data underlying the findings described in their manuscript fully available without restriction, with rare exception (please refer to the Data Availability Statement in the manuscript PDF file). The data should be provided as part of the manuscript or its supporting information, or deposited to a public repository. For example, in addition to summary statistics, the data points behind means, medians and variance measures should be available. If there are restrictions on publicly sharing data—e.g. participant privacy or use of data from a third party—those must be specified.

Reviewer #1: Yes

Reviewer #2: Yes

**********

4. Is the manuscript presented in an intelligible fashion and written in standard English?

PLOS ONE does not copyedit accepted manuscripts, so the language in submitted articles must be clear, correct, and unambiguous. Any typographical or grammatical errors should be corrected at revision, so please note any specific errors here.

Reviewer #1: Yes

Reviewer #2: Yes

**********

5. Review Comments to the Author

Please use the space provided to explain your answers to the questions above. You may also include additional comments for the author, including concerns about dual publication, research ethics, or publication ethics. (Please upload your review as an attachment if it exceeds 20,000 characters)

Reviewer #1: This is an interesting analysis and obviously timely. I have only one significant comment and it is a criticism but something that should be discussed. In the data presented on the Y1-Y2 differences in number of drinks the significant DID seems to be mediated by both a larger number of Y2 drinks and a smaller number of Y1 for the pandemic exposed individuals. Is this apparent difference statistically significant, what could have caused it and would controlling for it reduce the effect of the pandemic?

Reviewer #2: This manuscript uses a unique and large dataset in order to answer an important question - what is the impact of experiencing the COVID-19 pandemic on alcohol consumption and is this different for subgroups of people with known COVID-19 vulnerabilities? That cannot be overstated. However, there are a few concerns that I have with the manuscript that dampen enthusiasm.

- The alcohol consumption measures are limited to drinks per occasion and binge drinking. Validated measures of alcohol consumption or an index of alcohol use disorder are not included in this dataset. This should be stated as a limitation in the discussion.

- The 3rd paragraph of the discussion around the self-medication hypothesis is entirely and highly speculative given the data available and recommend removing entirely. I am not sure that perpetuation of a self-medication hypothesis in the substance use disorder field is helpful.

- The mental health measures used necessitate changing references to anxiety and depression to anxiety and depression symptoms as they are not diagnostic in nature.

- The data figures (3 and 4) are not legible and need to be improved.

**********

6. PLOS authors have the option to publish the peer review history of their article (what does this mean?). If published, this will include your full peer review and any attached files.

If you choose “no”, your identity will remain anonymous but your review may still be made public.

**Do you want your identity to be public for this peer review?** For information about this choice, including consent withdrawal, please see our Privacy Policy.

Reviewer #1: No

Reviewer #2: No

[NOTE: If reviewer comments were submitted as an attachment file, they will be attached to this email and accessible via the submission site. Please log into your account, locate the manuscript record, and check for the action link "View Attachments". If this link does not appear, there are no attachment files.]

While revising your submission, please upload your figure files to the Preflight Analysis and Conversion Engine (PACE) digital diagnostic tool, https://pacev2.apexcovantage.com/. PACE helps ensure that figures meet PLOS requirements. To use PACE, you must first register as a user. Registration is free. Then, login and navigate to the UPLOAD tab, where you will find detailed instructions on how to use the tool. If you encounter any issues or have any questions when using PACE, please email PLOS at figures@plos.org. Please note that Supporting Information files do not need this step.

# Author comment: Author response to Decision Letter 0

**DOI:** 10.1371/journal.pone.0266422.r002

13 Feb 2022

Response to reviews:

Reviewer #1: This is an interesting analysis and obviously timely. I have only one significant comment and it is a criticism but something that should be discussed. In the data presented on the Y1-Y2 differences in number of drinks the significant DID seems to be mediated by both a larger number of Y2 drinks and a smaller number of Y1 for the pandemic exposed individuals. Is this apparent difference statistically significant, what could have caused it and would controlling for it reduce the effect of the pandemic?

RESPONSE: This reviewer raises an important question that our collaborative group discussed at length when devising our analytic strategy. We followed a rigorous methodology consistent with difference-in-difference analyses to test model assumptions as recommended by Wing et al. (2018). The main assumption with these models is the “parallel trend assumption” which presupposes that the trend over time observed in the unexposed group would have been observed in our exposed group had they not been exposed. We used historical data collected in the TBI Model Systems from 2015-2016 (i.e., preceding the timeframe of our analytic sample) to test our parallel trend assumption for the primary analyses and all subgroup analyses (see Supplemental Figures 1-17). The main parameter of interest in difference-in-difference analyses is the interaction term (β3) in the general equation below. 

E(Y) = β0 + β1(Pandemic Exposed) + β2(Time) + β3(Exposed*Time) + ɛ

Crucially, while these longitudinal models do already account for the baseline main effects of exposure status (β1), it is not required that β1 be non-significant for there to be an observed difference between exposure groups over time (e.g., β3). Therefore, any small difference in average number of drinks at baseline (0.91 unexposed vs. 0.78 exposed) do not change the conclusions regarding the impact of the pandemic observed herein. To make this point clearer, we have now clarified in our Methods section that the parallel trend assumption does not presuppose that exposure groups be balanced with respect the outcome at baseline (p. 8 lines 205-208). We have also provided the reference below that provides further detail that may be of interest.

Reference:

Wing C, Simon K, Bello-Gomez RA. Designing difference in difference studies: best practices for public health policy research. Annual review of public health. 2018;39.

Reviewer #2: This manuscript uses a unique and large dataset in order to answer an important question - what is the impact of experiencing the COVID-19 pandemic on alcohol consumption and is this different for subgroups of people with known COVID-19 vulnerabilities? That cannot be overstated. However, there are a few concerns that I have with the manuscript that dampen enthusiasm.

- The alcohol consumption measures are limited to drinks per occasion and binge drinking. Validated measures of alcohol consumption or an index of alcohol use disorder are not included in this dataset. This should be stated as a limitation in the discussion.

RESPONSE: We thank this reviewer for this comment to allow us the opportunity to clarify details on the strengths and limitations of our alcohol data collection in this study. In 2017, the TBIMS National Database changed their data collection protocol to be consistent with the National Institute on Alcohol Abuse and Alcoholism (NIAAA) guidelines for binge drinking, which are 5+ drinks on an occasion for men, and 4+ drinks on an occasion for women. This NIAAA sex-specific binge drinking definition was applied in our full sample, which we have described in our methods section (p. 6 lines 149-151). That said, this reviewer’s point is well-taken, and unfortunately we did not have information available in this study on alcohol use disorder, nor measures related to consequences of drinking. We have now added these points as limitations in our discussion (p. 14 lines 360-362).

- The 3rd paragraph of the discussion around the self-medication hypothesis is entirely and highly speculative given the data available and recommend removing entirely. I am not sure that perpetuation of a self-medication hypothesis in the substance use disorder field is helpful.

RESPONSE: As suggested by the reviewer, we have removed discussion of the self-medication hypothesis in the discussion (p. 12 lines 307-310).

- The mental health measures used necessitate changing references to anxiety and depression to anxiety and depression symptoms as they are not diagnostic in nature.

RESPONSE: We thank this reviewer for this comment. We agree and have gone through our entire manuscript and made changes to the language such that anxiety and depression are now referred to as anxiety symptoms and depressive symptoms, respectively.

- The data figures (3 and 4) are not legible and need to be improved.

RESPONSE: We thank the reviewer for this feedback. We have now split up Figures 3 and 4 into multiple figures, which substantially reduces the number of panels in each figure. We have also enhanced the quality of each figure to further aid in legibility.

Attachment Submitted filename: Response to reviews FINAL.docx Click here for additional data file.

# Peer review: Decision Letter 1

**DOI:** 10.1371/journal.pone.0266422.r003

**Authors:** Samuel Wilkinson

21 Mar 2022

Changes in Alcohol Use and Mood during the COVID-19 Pandemic among Individuals with Traumatic Brain Injury: A Difference-in-Difference Study

PONE-D-21-37809R1

Dear Dr. Kumar,

We’re pleased to inform you that your manuscript has been judged scientifically suitable for publication and will be formally accepted for publication once it meets all outstanding technical requirements.

Within one week, you’ll receive an e-mail detailing the required amendments. When these have been addressed, you’ll receive a formal acceptance letter and your manuscript will be scheduled for publication.

An invoice for payment will follow shortly after the formal acceptance. To ensure an efficient process, please log into Editorial Manager at http://www.editorialmanager.com/pone/, click the 'Update My Information' link at the top of the page, and double check that your user information is up-to-date. If you have any billing related questions, please contact our Author Billing department directly at authorbilling@plos.org.

If your institution or institutions have a press office, please notify them about your upcoming paper to help maximize its impact. If they’ll be preparing press materials, please inform our press team as soon as possible -- no later than 48 hours after receiving the formal acceptance. Your manuscript will remain under strict press embargo until 2 pm Eastern Time on the date of publication. For more information, please contact onepress@plos.org.

Kind regards,

Samuel Wilkinson, MD

Academic Editor

PLOS ONE

Additional Editor Comments (optional):

Reviewers' comments:

Reviewer's Responses to Questions

**Comments to the Author**

1. If the authors have adequately addressed your comments raised in a previous round of review and you feel that this manuscript is now acceptable for publication, you may indicate that here to bypass the “Comments to the Author” section, enter your conflict of interest statement in the “Confidential to Editor” section, and submit your "Accept" recommendation.

Reviewer #1: All comments have been addressed

**********

2. Is the manuscript technically sound, and do the data support the conclusions?

The manuscript must describe a technically sound piece of scientific research with data that supports the conclusions. Experiments must have been conducted rigorously, with appropriate controls, replication, and sample sizes. The conclusions must be drawn appropriately based on the data presented. 

Reviewer #1: Yes

**********

3. Has the statistical analysis been performed appropriately and rigorously? 

Reviewer #1: Yes

**********

4. Have the authors made all data underlying the findings in their manuscript fully available?

The PLOS Data policy requires authors to make all data underlying the findings described in their manuscript fully available without restriction, with rare exception (please refer to the Data Availability Statement in the manuscript PDF file). The data should be provided as part of the manuscript or its supporting information, or deposited to a public repository. For example, in addition to summary statistics, the data points behind means, medians and variance measures should be available. If there are restrictions on publicly sharing data—e.g. participant privacy or use of data from a third party—those must be specified.

Reviewer #1: Yes

**********

5. Is the manuscript presented in an intelligible fashion and written in standard English?

PLOS ONE does not copyedit accepted manuscripts, so the language in submitted articles must be clear, correct, and unambiguous. Any typographical or grammatical errors should be corrected at revision, so please note any specific errors here.

Reviewer #1: Yes

**********

6. Review Comments to the Author

Please use the space provided to explain your answers to the questions above. You may also include additional comments for the author, including concerns about dual publication, research ethics, or publication ethics. (Please upload your review as an attachment if it exceeds 20,000 characters)

Reviewer #1: (No Response)

**********

7. PLOS authors have the option to publish the peer review history of their article (what does this mean?). If published, this will include your full peer review and any attached files.

If you choose “no”, your identity will remain anonymous but your review may still be made public.

**Do you want your identity to be public for this peer review?** For information about this choice, including consent withdrawal, please see our Privacy Policy.

Reviewer #1: No

# Editor report: Acceptance letter

**DOI:** 10.1371/journal.pone.0266422.r004

**Authors:** Samuel Wilkinson

24 Mar 2022

PONE-D-21-37809R1 

Changes in alcohol use and mood during the COVID-19 pandemic among individuals with traumatic brain injury: A difference-in-difference study 

Dear Dr. Kumar:

I'm pleased to inform you that your manuscript has been deemed suitable for publication in PLOS ONE. Congratulations! Your manuscript is now with our production department. 

If your institution or institutions have a press office, please let them know about your upcoming paper now to help maximize its impact. If they'll be preparing press materials, please inform our press team within the next 48 hours. Your manuscript will remain under strict press embargo until 2 pm Eastern Time on the date of publication. For more information please contact onepress@plos.org.

If we can help with anything else, please email us at plosone@plos.org. 

Thank you for submitting your work to PLOS ONE and supporting open access. 

Kind regards, 

PLOS ONE Editorial Office Staff

on behalf of

Dr. Samuel Wilkinson 

Academic Editor

PLOS ONE
